# A High Performance, Cost-Effective, Open-Source Microscope for Scanning Two-Photon Microscopy that Is Modular and Readily Adaptable

**DOI:** 10.1371/journal.pone.0110475

**Published:** 2014-10-21

**Authors:** David G. Rosenegger, Cam Ha T. Tran, Jeffery LeDue, Ning Zhou, Grant R. Gordon

**Affiliations:** 1 Department of Physiology and Pharmacology, Cumming School of Medicine, University of Calgary, Hotchkiss Brain Institute, Calgary, Alberta, Canada; 2 Department of Psychiatry, University of British Columbia, Brain Research Centre, Vancouver, British Columbia, Canada; 3 Graduate Institute of Clinical Medical Science, China Medical University, Translational Medicine Research Center, Taichung, Taiwan; University of Zurich, Switzerland

## Abstract

Two-photon laser scanning microscopy has revolutionized the ability to delineate cellular and physiological function in acutely isolated tissue and *in* *vivo*. However, there exist barriers for many laboratories to acquire two-photon microscopes. Additionally, if owned, typical systems are difficult to modify to rapidly evolving methodologies. A potential solution to these problems is to enable scientists to build their own high-performance and adaptable system by overcoming a resource insufficiency. Here we present a detailed hardware resource and protocol for building an upright, highly modular and adaptable two-photon laser scanning fluorescence microscope that can be used for *in* *vitro* or *in* *vivo* applications. The microscope is comprised of high-end componentry on a skeleton of off-the-shelf compatible opto-mechanical parts. The dedicated design enabled imaging depths close to 1 mm into mouse brain tissue and a signal-to-noise ratio that exceeded all commercial two-photon systems tested. In addition to a detailed parts list, instructions for assembly, testing and troubleshooting, our plan includes complete three dimensional computer models that greatly reduce the knowledge base required for the non-expert user. This open-source resource lowers barriers in order to equip more laboratories with high-performance two-photon imaging and to help progress our understanding of the cellular and physiological function of living systems.

## Introduction

Two-photon laser scanning microscopes have clear advantages over visible light confocal and deconvolution systems, as they are able to image deep within highly light scattering tissues, have inherent optical sectioning, and limit damage to cells [1], [2]. Many laboratories though are unable to acquire such useful microscopes due to various barriers, including a lack of detailed open-source resources that enable non-expert users to execute on do-it-yourself endeavors. Additionally, if a microscope is possessed, most commercial and some custom platforms are difficult to adapt to rapidly changing technologies.

Custom-built scanning microscopes for two-photon imaging have existed in laboratories for more than two decades [3], [4], [5], [6], [7], [8]. While there have been numerous developments to enhance performance [4], [9], [10], [11], [12], [13], [14], [15], [16], there are only a few hardware platforms on which custom systems are based. First, a two-photon capable system can be made by retrofitting a commercial, visible laser scanning confocal microscope [5], [17], [18], [19], [20]. One advantage of this technique is that the microscope already possesses a scan head as well as optics and camera ports for other types of light microscopy. The disadvantages are the need of an expensive confocal microscope and the end product after retrofitting remains difficult to adapt. Second, a custom two-photon laser-scanning microscope can be built by assembling individual opto-mechanical elements onto rails using parts from Thorlabs, Newport or Edmund Optics. The advantage of this platform is that it is cost-effective, modular and adaptable due to the availability and compatibility of the different parts. The clear disadvantages are the necessary microscopy knowledge base required for the initial design, and the lack of detailed resources for the construction of a high performance system (though see [7]). Furthermore, typical opto-mechanical systems lack a transmitted light path for bright field microscopy; thus limiting their application. Finally, custom two-photon designs are freely available from Janelia Farms at *openwiki.janelia.org*, from an open source lab tech website called *labrigger.com* and from the Parker Lab at UC Irvine at http://parkerlab.bio.uci.edu. The Janelia systems are specialized for *in* *vivo* experiments, and are a combination of opto-mechanical parts and custom designed machined components. The advantages are that a detailed resource is provided and the dedicated design makes these systems high performance for their intended use. The disadvantages are that the overall design requires numerous custom machined parts, making the system less flexible compared to systems based primarily on off-the-shelf opto-mechanical parts. In summary, a combination of these different platforms is needed.

Here we provide a detailed open-source hardware plan for constructing a two-photon laser scanning microscope we call TIMAHC (pronounced *tie-mac*) which stands for Two-photon Imaging that is Modular, Adaptable, High-performance and Cost-effective. TIMAHC combines many of the strengths of the different custom microscope strategies described above: 1) TIMAHC is built almost entirely out of off-the-shelf compatible opto-mechanical parts from Thorlabs to which high-end componentry can be easily fastened and adapted; 2) The purpose-selected hardware, along with simple yet effective light paths, allow for deep imaging (nearing 1 mm in highly light scattering tissue) and an excellent signal-to-noise ratio; 3) To overcome a microscope building knowledge gap, we provide 3D digital models (viewed in freely available SolidWorks eDrawings), a complete parts list, building instructions, as well as tips for testing and troubleshooting; 4) In addition to the hardware required for scanning, motorization, and two-channel fluorescence collection (detailed below), TIMAHC has an under-stage PMT for transmitted laser light detection and an incoherent Koehler Illumination setup for tissue visualization; 5) TIMAHC is optimized for work on acutely isolated tissue but can be adapted within minutes for *in* *vivo* two-photon imaging. Finally, although this resource is intended to facilitate the non-expert user in building a custom two-photon imaging system, it must be stressed that such an endeavour is a significant undertaking.

## Methods and Results: Microscope Build

**Figure 1:**
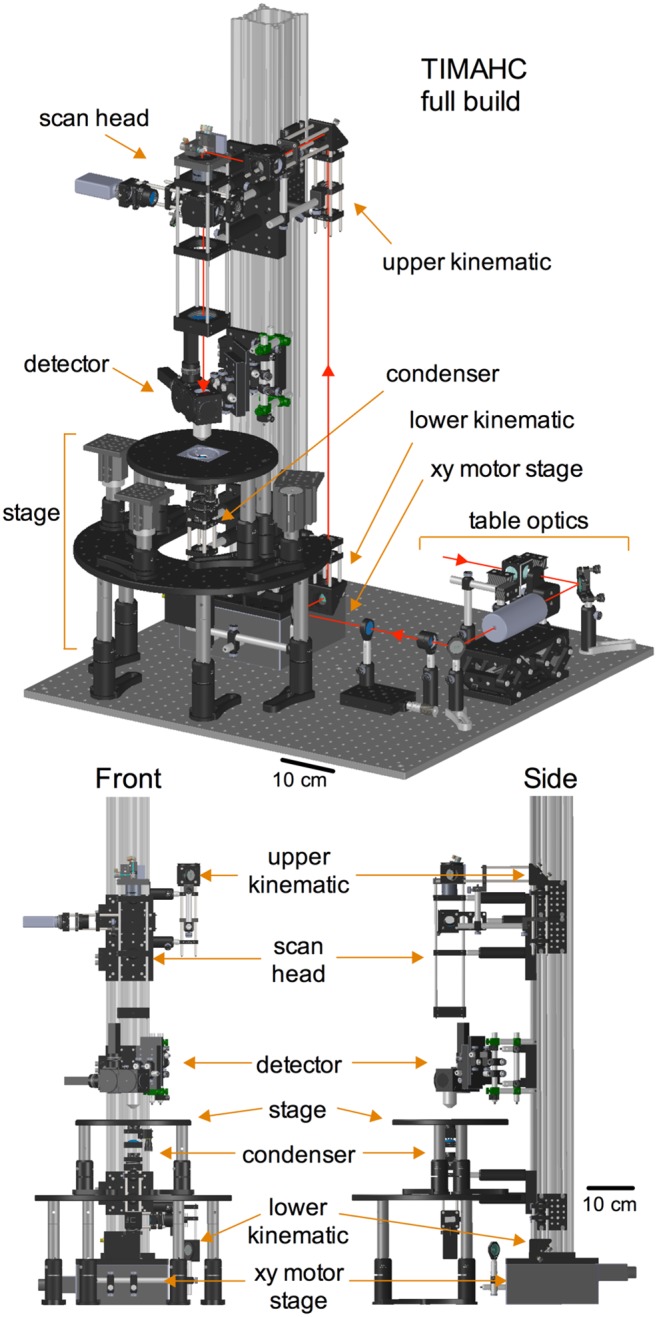
TIMAHC full build. All the sub-assemblies are indicated including: detector sub-assembly, scanning sub-assembly, condenser sub-assembly, kinematic sub-assembly, xy motor stage sub-assembly, sample stage sub-assembly and table optics sub-assembly. Red line indicates the path of the Ti:Sapph laser. Angled perspective view (top), front view (left) and side view (right) is shown (see Model S1– Microscope Assembly).

Our objective was to build a high performance microscope for laser scanning two-photon fluorescence imaging which could be used for both *in* *vitro* and *in* *vivo* applications, and also be combined with other methodologies such as visually guided patch clamp electrophysiology. A primary aim was to make the microscope as flexible and affordable as possible without the need for costly and time-consuming custom machining. These goals lead to the creation of TIMAHC (Fig. 1; Model S1 - Microscope Assembly, viewed using SolidWorks eDrawings; Table S1 - complete parts and price list).

### Software control and microscope hardware

TIMAHC is intended to be paired with existing and widely adopted open-source control software. TIMAHC has been thoroughly tested with ScanImage [21] (*openwiki.janelia.org*). ScanImage runs in Matlab and makes use of control and acquisition hardware from National Instruments. A compatible microscope hardware list is provided in the ScanImage documentation. As such, TIMAHC incorporates 5 mm galvanometric scanners (Cambridge Tech, part# 6210H), GaAsP PMT detectors (Hamamatsu, part# H10770PA-40), Pockel’s cells for fast laser power control (Conoptics, part# 350-80LA), and xyz motorization (Sutter, part# MP-285; Dover Motion, part# XYR-8080). TIMAHC may also be controlled with other open source software platforms such as Helioscan [22] or MPScope [23] but these platforms have not been tested.

### Detector sub-assembly

**Figure 2:**
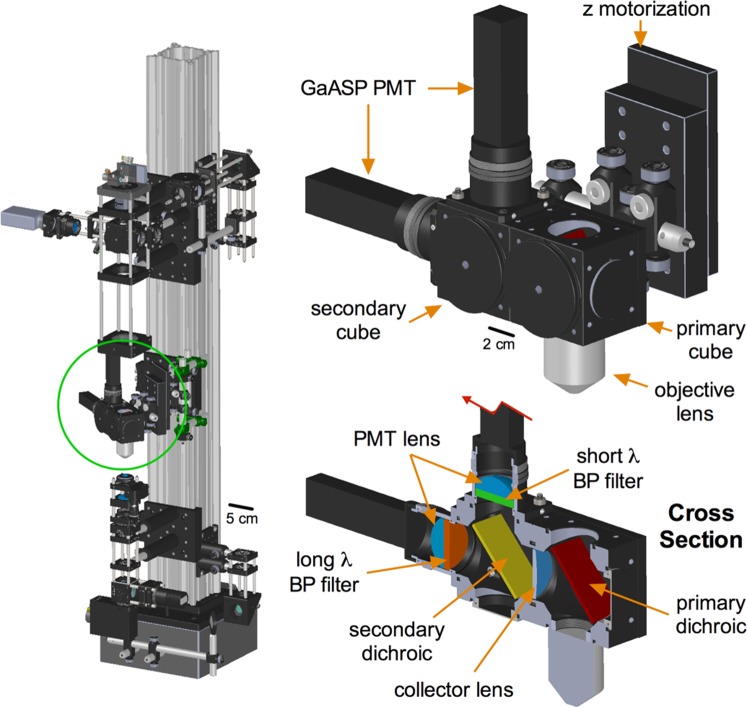
The detector sub-assembly. **Left**) Green circle localizes the detector sub-assembly on TIMAHC. **Top**) Main parts indicated including: GaAsP PMTs, fluorescence cubes, objective lens and z motorized slider. **Bottom**) Cross section showing the internal optics: primary and secondary dichroics, green and red emission filters, primary collection lens and PMT aspheric lenses (see Model S1– detector sub-assembly).

The detector sub-assembly (Fig. 2) was designed with a short (13.5 cm), fixed length, fluorescence collection path, measured from the back aperture of the objective lens to the PMT detectors. This was achieved by connecting the entire detector assembly, including the objective lens, to the z motorization stepper (Sutter, part# MP-285). The design principle was to minimize florescence signal loss caused by the failure of a long collection light path to capture scattered (non-ballistic) photons [1], [24]. The detector sub-assembly incorporates two GaAsP PMT detectors (Hamamatsu, part# H10770PA-40), which have greater sensitivity than standard bialkali or multialkali PMTs. To direct the emitted light, we used custom size fluorescence optics and standard collection optics. First, the primary dichroic mirror (Chroma, part# 695cxxr) allows passage of the Ti:Sapph beam to the sample, while reflecting epi-fluorescence light shorter than 695 nm towards the detectors. Once reflected, a closely apposed 1″ collector lens (Thorlabs, part# LA1708-A) focuses the fluorescence light towards the secondary dichroic mirror. We use a green/red splitter (Chroma, part# T560LPXR) but an alternative dichroic with corresponding emission filters can be used as desired. The divided light then travels on separate paths towards either a green (Chroma, part# ET525/50m-2P) or an orange/red (ET605/70m-2P) emission filter before passing through a final aspheric lens (Thorlabs, part# LA1805-A) that focuses the light on to the GaAsP PMT. TIMAHC allows the use of high numerical aperture (NA) 0.8–1.1 water dipping objective lenses. We have thoroughly tested the use of a 40X, NA1.0 lens (Zeiss, part# 441452990), which has a steep access angle and a good working distance (2.5 mm) for patch pipettes. Alternative objective lenses can be used as desired, taking into consideration the max beam diameter possible on TIMAHC and the diameter of the objective lens back aperture (see scanning sub-assembly and discussion). For each objective lens, an adapter collar must be purchased in order to couple the lens with the opto-mechanics (Thorlabs: optical component thread adapters). Magnetic collars can be added to ease the changing of objective lenses (not shown in model, Thorlabs, part# CP90f). Finally, the motorized z-axis slider (Sutter, part# MP-285-1z, high load bearing) provides 1″ of travel to the objective lens and can move in increments of 0.1 µm for z-stack acquisitions (Model S1 - detector sub-assembly; see Figure S1 for a detector mounting alignment aid).

### Scanning sub-assembly

**Figure 3:**
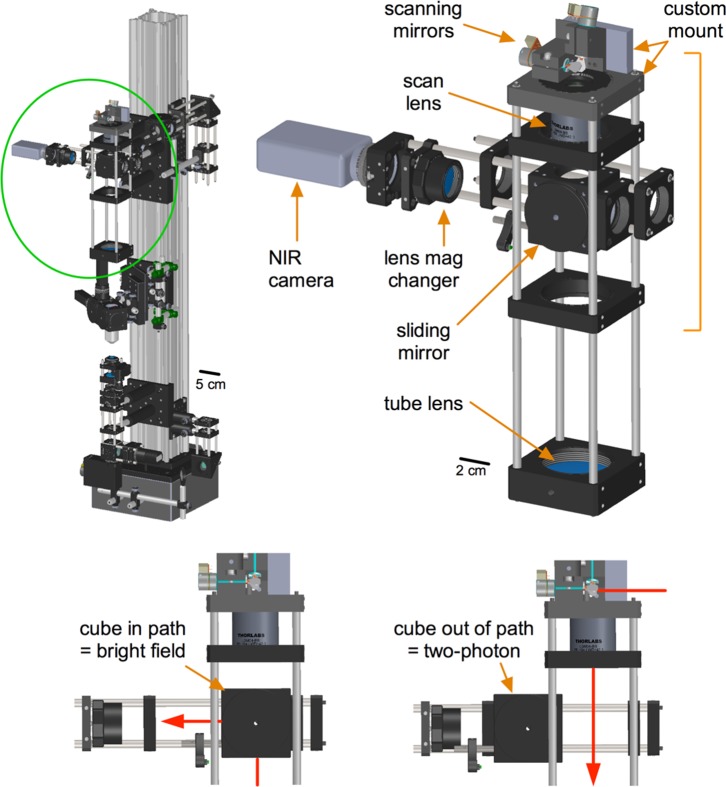
The scanning sub-assembly. **Left**) Green circle localizes the scanning sub-assembly on TIMAHC. **Right**) Main parts of the scanning sub-assembly indicated including: galvanometric scanning mirrors, scan lens, tube lens, sliding cube, lens mag changer and NIR camera. **Bottom**) Different positions of the sliding mirror cube are shown to select for either two-photon imaging or a LED/camera transmitted image of the sample (see Model S1– scanning sub-assembly).

TIMAHC’s scanning sub-assembly (Fig. 3) incorporates mounted galvanometric scanners (Cambridge Tech, part# 6210H) that direct the Ti:Sapph beam first into a scan lens (Thorlabs, part# LSM04-BB) and then through a doublet achromatic tube lens (Thorlabs, AC508-200-B). These two lens elements are separated from each other by the sum of their focal lengths (∼254 mm). The lenses cause a beam diameter expansion of 3.7 times. The scanning mirrors on TIMAHC can accept a beam diameter up to 5 mm and given the expansion ratio of 3.7 above, the largest beam arriving at the back aperture of the objective lens is 18.5 mm. This permits the use of low magnification, high NA objective lenses that have a large back aperture.

In the scanning sub-assembly, TIMAHC has a manual sliding mirror cube and hardware to mount a near infrared (NIR) sensitive camera for tissue visualization (Dage MTI, part #IR-1000). When the sliding mirror is positioned on the optical axis, it directs the transmitted light from the under-stage 940 nm LED to the camera. When the sliding cube is moved out from the optical axis, it allows the Ti:Sapph beam to travel down the optical axis of the microscope for two-photon fluorescence imaging. This configuration was chosen due to the fact that both the LED and the Ti:Sapph outputs overlap in the NIR wavelength range, making a fixed (non sliding) dichroic mirror untenable. In between the sliding mirror cube and the camera we incorporated a magnet collar system so that lenses of different focal lengths can be easily added or removed from the light path to provide different magnifications of the transmitted image without switching objective lenses. The absence of a lens in the magnet collar provides the largest magnification whereas a lens of focal length ∼150 mm (Thorlabs, part# AC254-150-B) provides the full field of view. For a low cost the user can add lenses for desired magnifications (Model S1 - scanning sub-assembly).

### Condenser sub-assembly

**Figure 4:**
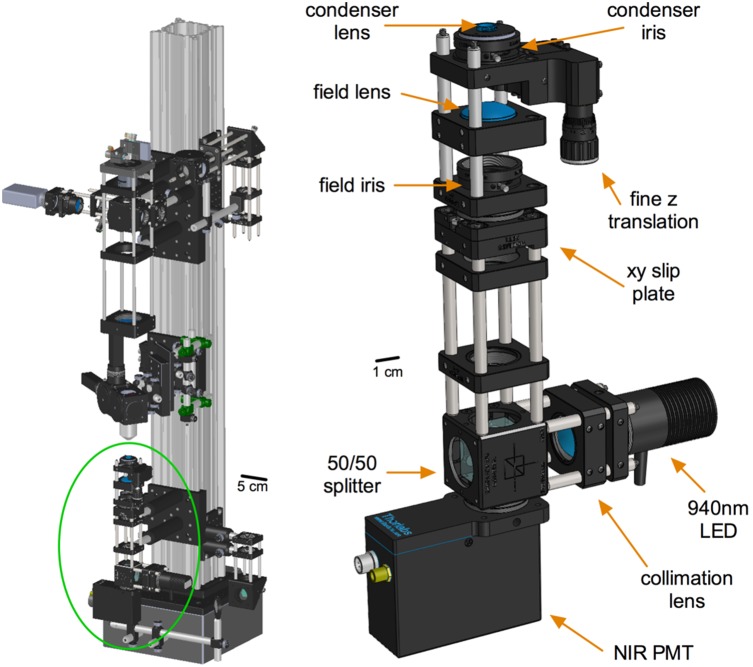
The condenser sub-assembly. **Left**) Green circle localizes the condenser sub-assembly on TIMAHC. **Right**) Main parts of the condenser are shown including: condenser lens, field lens, condenser iris, field iris, fine z translator, 50/50 splitter cube, collimation lens, 940 nm LED and NIR sensitive PMT (see Model S1– condenser sub-assembly).

TIMAHC incorporates a condenser (Fig. 4) with components for Koehler Illumination, similar to that found on numerous light microscopes. There is a field iris (Thorlabs, part# SM1D12SS) to control the size of the viewing area and to help guide condenser centering. A condenser iris controls the NA of the transmitted image as well as the brightness and contrast. There is a high NA condenser lens (0.69) (Thorlabs, part# C330TME-B) that enables a crisp transmitted image of the sample down to fields of view of approximately 100 microns. The condenser lens is mounted within a z-translation element (Thorlabs, part# SM1Z) that allows fine adjustment of the height in order to make the image plane conjugate with the field iris. An additional xy slip plate (Thorlabs, part# SPT1) allows the user to center the field iris, and thus the condenser lens, to the optical axis to complete the Koehler setup. TIMAHC makes use of a NIR (940 nm) LED light source (Thorlabs, part# M940L3) that can clearly image thick, light scattering tissue samples like acutely isolated brain slices. Furthermore, the entire condenser assembly (along with the stage) can be easily lowered to increase the z-axis space under the objective lens for *in* *vivo* experiments. If large *in* *vivo* preparations are desired, the condenser assembly can be completely removed and by switching to shorter stage posts much more space can be achieved. Finally, the condenser assembly incorporates an under-stage NIR-sensitive PMT (Thorlabs, part# PMM02) to capture the transmitted image of the tissue generated by the Ti:Sapph beam. Detection of transmitted wavelengths up to 950 nm are possible (Model S1 - condenser sub-assembly).

### Table optics sub-assembly

**Figure 5:**
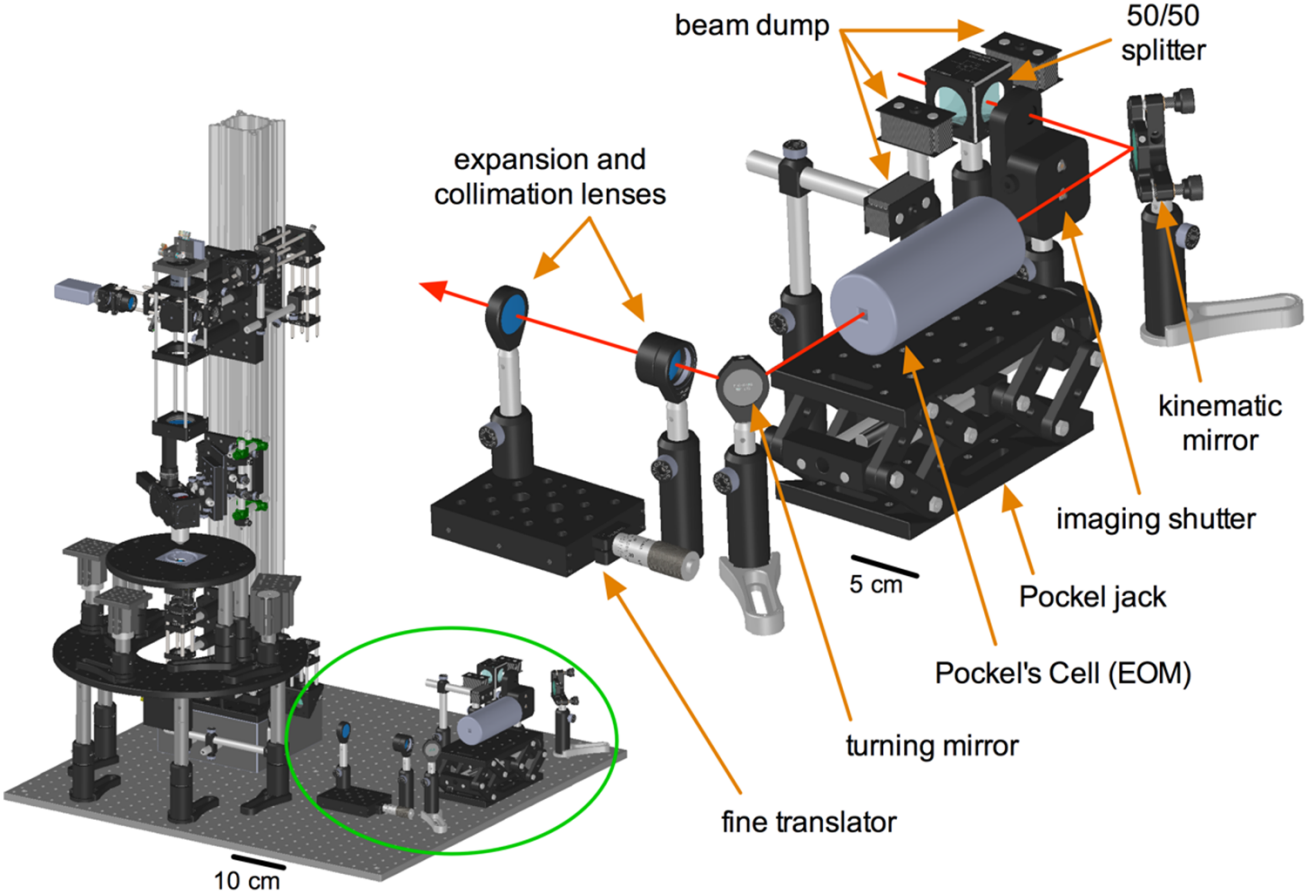
Table optics sub-assembly. **Left**) Green circle localizes the table optics sub-assembly in relation to TIMAHC. **Top**) Main components of the sub-assembly are shown including: 50/50 beam splitter, imaging shutter, beam dumps, kinematic turning mirror, Pockel’s cell and stand, fixed turning mirror(s) as well as beam expansion and collimation parts (two achromatic doublet lenses and a translator). Red line indicates the path of the Ti:Sapph laser through the table optics (see Model S1– table optics sub-assembly).

For the Ti:Sapph beam to be controlled and directed into the microscope, various table optics are required (Fig. 5). Out of the laser head, the Ti:Sapph beam first encounters a 50/50 beam splitter (Thorlabs, part# CM1-BS015). This is to half the power of the beam going to TIMAHC because power is typically in excess, or to split the beam to feed another experimental setup. Next in the beam path is the imaging shutter (Thorlabs, part# SH05), which opens and closes in correspondence with the beginning and end of an image acquisition. After the shutter we send the beam to a kinematic mirror that deflects the beam at an adjustable angle (Thorlabs, part# KM100). The kinematic mirror is necessary to fine-tune the downstream path of the beam along the table through the various optics. Next is a Pockel’s cell or electro-optic modulator (Conoptics, part# 350-80LA) for microsecond fast power control of the laser beam. The Pockel’s cell provides easy, live adjustment of the laser power while imaging, but most importantly it is needed for experiments that involve rapid photo-stimulation such as uncaging [25] or bleaching events such as FRAP [26]. The beam then travels through a pair of collimating and beam expanding doublet-achromatic convex lenses (Thorlabs, part# AC254-30-B and -60-B). The two lenses are separated by the sum of their focal lengths (90 mm) and the focal length ratio dictates the degree of expansion (2X). This pair of lenses is required to create an appropriate beam diameter to be received by the scanning mirrors and to make fine adjustments in collimation by the use of a translating axis (Thorlabs, part# PT1). Collimation is critical when the beam enters into the back aperture of the objective so that the lens works as intended. Beam diameter is also critical to fill the back aperture of the objective to realize the NA. Because the beam has a Gaussian profile, this is achieved where at least 1/*e^2^* of the beam intensity equals the diameter of the back aperture [1], [8]. After expansion and collimation, the beam enters into the on-scope kinematic sub-assembly. Note that additional standard (non-kinematic) mirror mounts can be added to any point on the table beam path when a 90-degree turn is desired. We use broadband dielectric mirrors on the table and in the microscope (Thorlabs, part# BB1-E03) (Model S1 - table optics sub-assembly).

### Kinematic sub-assembly and xy motorization

**Figure 6:**
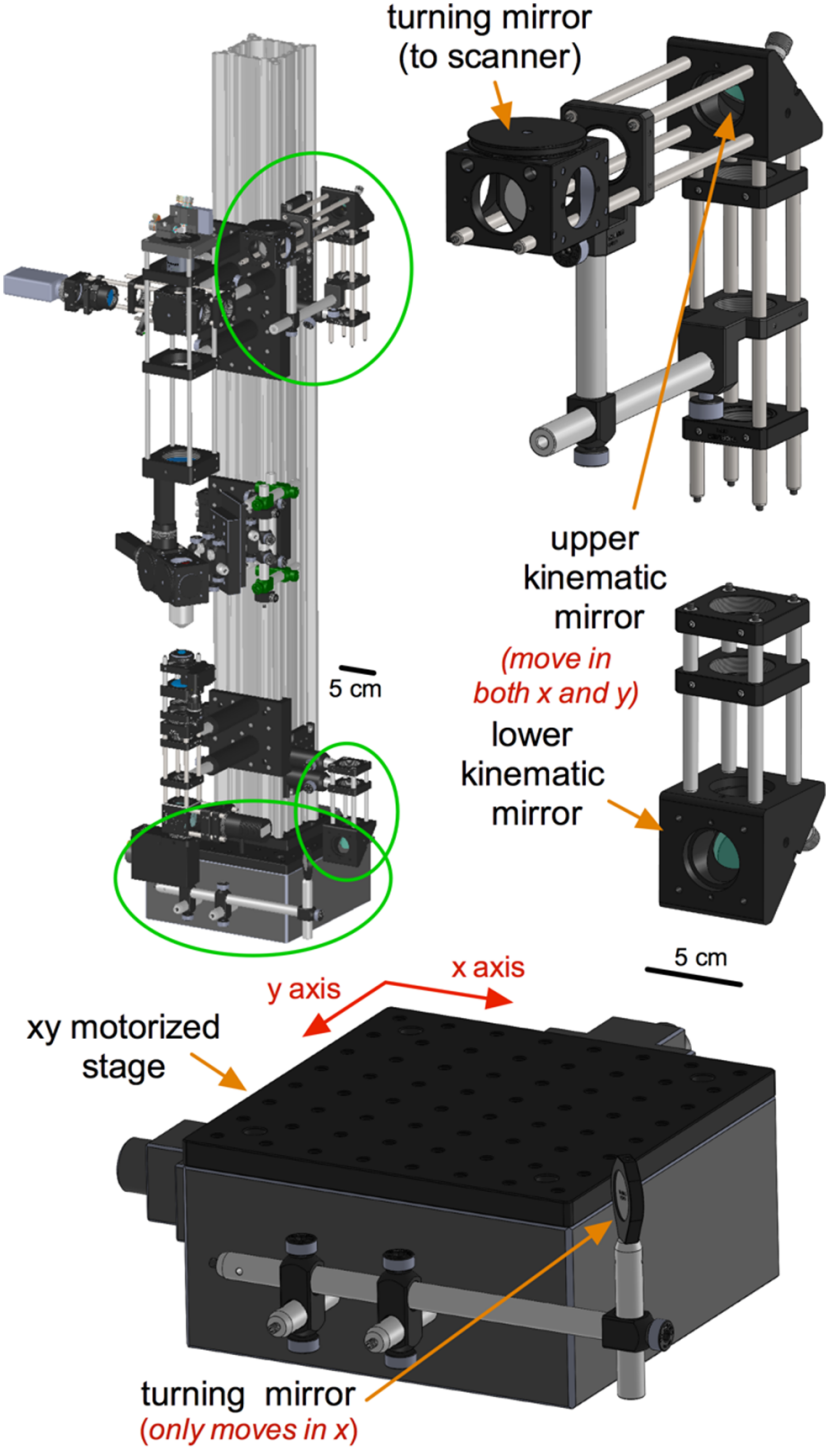
The kinematic mirrors and xy motor stage sub-assemblies. **Left**) Green circles localize the kinematic mirrors and xy motor stage sub-assemblies on TIMAHC. **Right**) Upper and lower kinematic mirrors are shown, along with a turning mirror that directs the beam onto the scanning mirrors. **Bottom**) xy motor stage and single-axis turning mirror are shown. This allows TIMAHC to move in the xy direction while maintaining beam alignment (see Model S1– kinematic sub-assembly).

TIMAHC is relatively unique in the sense that the microscope itself moves in the x and y direction and the stage - where the *in* *vitro* sample or whole animal is positioned - is stationary (Fig. 6). This is advantageous for *in* *vivo* setups in which the animal cannot be moved during experimentation. The main optical rail of TIMAHC mounts to a motorized xy translational platform (Dover Motion, part# XYR-8080). This device can bear a high load (∼125 kg) and has a large range of motion in the x and y direction (15 cm for each axis). Notably, the device can be moved to such great extents that the condenser sub-assembly can contact the sample stage. To avoid this, we thread steel posts (Thorlabs, part# TR2 and TR3) into the optical table surrounding the XYR-8080 to limit the xy translation. Beam guidance mirrors are mounted to the translational axes on the XYR-8080 in such a way that maintain beam alignment when the microscope moves. The first of these is a turning mirror mounted to the front of the XYR-8080, which only moves in the x direction. The second and third guidance mirrors are mounted in the lower and upper kinematic sub-assemblies, which move in both the x and y direction. The kinematic mirrors are used to precisely guide the Ti:Sapph beam onto the galvanometric scanning mirrors and down through the optical axis (Model S1 - kinematic sub-assembly and xy stage sub-assembly).

### Computer and power supplies

TIMAHC runs on a common computer: a 3.5 GHz quad core, 16 GB of RAM, a 500 W power supply and a 2 TB hard drive. We use a rack mountable 4 U computer from Superlogics. We refrain from specifying the exact motherboard and components because computer technology changes rapidly. The user must ensure that the motherboard utilized possesses the appropriate expansion slots to accept the National Instruments cards required by the open-source software platform (i.e. ScanImage). The 4 U computer chassis provides adequate room for the large control/acquisition card (National Instruments, part# PCI-6110). Additionally, several microscope components require linear power supplies including the scanning mirrors, the pre-amplifiers for the GaAsP PMT signals, the GaAsP PMTs and the under-stage PMT. We use generic linear power supplies to power these elements (Topward, parts# 6303D and 3815D), with the exception of purpose-built power supplies for the GaAsP PMTs (Hamamatsu, part# C7169). The power supplies for the PMTs also provide the gain control.

### Stage sub-assembly

**Figure 7:**
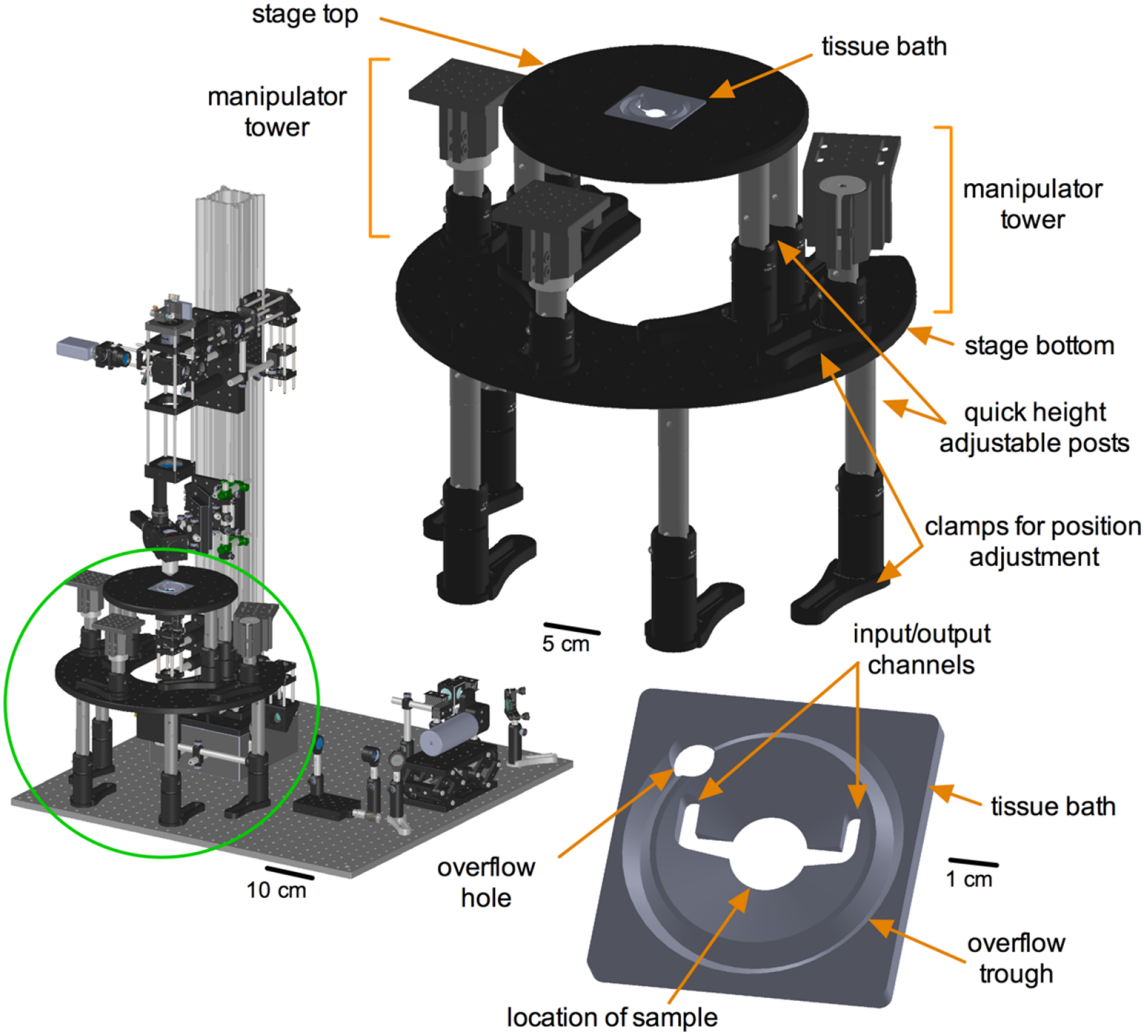
Sample stage sub-assembly. **Left**) Green circle localizes the sample stage sub-assembly in relation to TIMAHC. **Right**) Main parts of the stage sub-assembly are shown including: stage top, stage bottom, tissue bath, height adjustable legs and manipulator towers. **Bottom**) Close up of tissue bath is shown, highlighting sample compartment, input/output channels, over flow trough and over flow drainage hole (see Model S1– stage sub-assembly).

We designed a custom sample stage that allows fast and easy adjustments to changing experimental needs (Fig. 7). On the microscope itself there are only two custom machining jobs to be performed (both for the scanning sub-assembly scanning mirror mount). The custom sample stage necessitates an additional three pieces: the stage top, the stage bottom and a custom tissue bath. Both the stage top and the stage bottom are quickly height adjustable and can achieve a configuration for *in* *vivo* experiments within minutes. The stage top sports numerous tapped holes to fasten small manual manipulators, in-line heaters or other items needed for an experiment. The bottom stage provides a platform for the manipulator towers. Both the position of the towers on the bottom stage and the height of the towers can be readily adjusted for flexibility. Finally, the tissue bath was designed for acute brain slices but many other tissue types are possible within the spatial limits of the bath. There are input and output ports for buffer solution as well as an overflow trough and tube connection hole. These latter helps avoid solution spills that can ruin the under-stage irises, LED and PMT (Model S1 - stage sub-assembly).

### Additional Costs

TIMAHC is an affordable and flexible solution for a scanning microscope (∼$65 K USD), however, a typical two-photon laser light source is expensive. The most common is a Ti:Sapph laser, which is a broadly tunable (∼690–1040 nm), powerful (∼3 W) ultra-fast oscillator (∼80 MHz). Depending on the model, the cost range is $140–220 K USD. More affordable alternatives with fixed wavelength outputs (e.g. at 780 or 1040 nm) and less power are also available, as many fluorophores can still be excited. We use a Coherent Chameleon Ultra II (4 W avg power, 140 fs long pulses, 80 MHz, 670–1080 nm). For labs that cannot obtain a fluorescence imaging light source, we provide a 3D model detailing a modified version of TIMAHC meant for infrared transmitted imaging and photo-stimulation (Model S2 - IMEPS). Finally, any scanning microscope requires a vibration isolation table ($5–15 K). The degree of vibration isolation required (table top size, core and dampening) needs to be assessed in the location where the system is to be housed (consult the vendor). At a minimum, TIMAHC and a Ti:Sapph laser will require a tabletop surface area of 4×4 feet and be at least 8 inches thick (1.2 m×1.2 m×210 mm). We use a Newport S-2000 series with dimensions 4×8 feet and 8 inches thick (1.2 m×2.5 m×210 mm). The larger surface area is used to split the Ti:Sapph beam and feed another imaging system on the same table.

## Methods and Results: Microscope Performance

### Microscope Technical Performance

**Figure 8:**
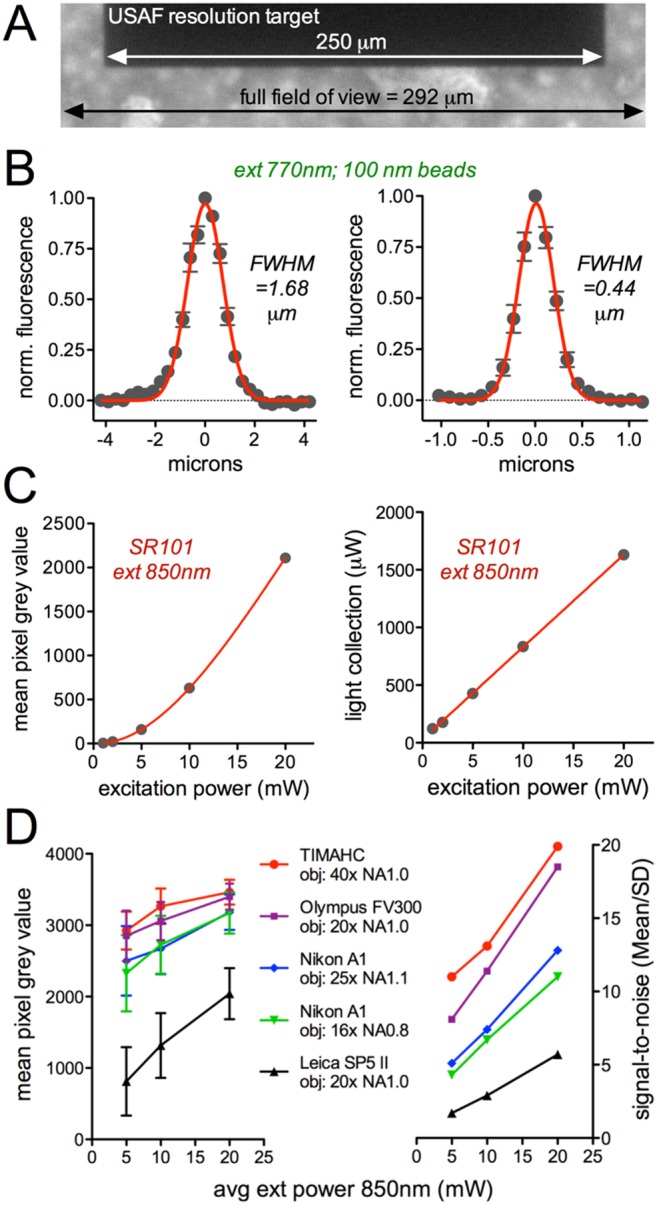
Microscope Performance: Field, resolution and signal-to-noise. **A**) The field of view using the Zeiss 40X NA1.0 objective at a scan angle of 15 degrees was determined by imaging a USAF standard resolution target. The dark bar is from group 1 element 1 on the target, which is 250 microns wide. The field was calculated to be 292 microns wide. **B**) Point-spread functions showing the axial (left) and radial (right) resolution limits determined by imaging 100 nm Fluosphere beads at 770 nm excitation. **C**) Fluorescence collection determined either by the PMT generated grey values on a 12 bit image at 60% detector gain (left) or measuring the power of the collected light with a photodiode power meter (right) when exciting the red dye SR-101 at different excitation powers. **D**) The mean red fluorescence signal and associated SD (left) and the signal to noise ratio: mean/SD (right) of SR-101 imaged at different powers of excitation. Data compares TIMAHC to three commercial two-photon systems: Olympus FV300, Nikon A1 and Leica SP5 II.

Using a USAF resolution target standard (Edmund Optics, cat# 58–198), we determined the size of the field of view imaged by TIMAHC. Using the Zeiss 40X NA1.0 lens with a scan angle of 15 degrees, we measured a field of view of 292^2^ µm (Fig. 8a). Next we examined the resolution limit. Using 100 nm diameter green Fluospheres (Life Technologies,# F-8803) and imaging at 770 nm, we found that TIMAHC had a radial (xy) resolution of 0.440 µm and an axial (z) resolution of 1.680 µm (full width half maximum (FWHM) of Gaussian fit, n = 8 beads, Fig. 8b). When compared to the theoretical resolution limit of 0.385 µm for radial and 1.540 µm for axial at this wavelength, TIMAHC performed close to diffraction limited. There was also no significant spherical aberration as the axial point spread function closely adhered to an expected Gaussian fit [27] (Fig. 8b left). Next we examined the collection efficiency of the system. At different powers of excitation light (detector gain at 60%), we imaged a solution of SR-101 (100 µM, Sigma, #S7635) at 850 nm and collected red fluorescence from 570–640 nm. We either measured the fluorescence signal detected with a PMT (Fig. 8c left) or used a photodiode power meter (Thorlabs, part# S121C), which was directly threaded onto the detector sub-assembly by replacing a PMT (1 mW excitation = 122 µW collected, 2 mW = 178 µW, 5 mW = 427 µW, 10 mW = 834 µW, 20 mW = 1630 µW, Fig. 8c right).

We then analyzed the signal-to-noise ratio on TIMAHC and compared it to three commercial two-photon imaging systems: Olympus FV300, Nikon A1 and Leica SP5 II. To do this, we kept the excitation wavelength (850 nm), excitation power (5, 10 and 20 mW), pixel dwell time or frame rate (∼3.2 µs or 1 Hz), field of view (∼150 µm), pixel density (512^2^), and image bit depth (12 bit) constant between the different microscopes. All microscopes used a Coherent Chameleon laser as the light source, thus keeping pulse width and repetition rate constant as well. We noted the objective NA (range 0.8 to 1.1) and imaged a solution of SR-101 (100 µM). For a given image, we made the brightest pixel as close as possible to the saturation point (grey value of 4096) by increasing detector gain. We calculated the mean signal over the standard deviation (SD) of the signal for every image. We found that TIMAHC had signal-to-noise characteristics that exceeded those of the commercial microscopes tested (S-to-N at 5, 10 and 20 mW: TIMAHC obj 40x NA1.0 = 11.0, 13.1, 19.9; Olympus FV300 obj 20x NA1.0 = 8.1, 11.4, 18.5; Nikon A1 obj 25x NA1.1 = 5.1, 7.4, 12.8; Nikon A1 obj 16x NA0.8 = 4.3, 6.7, 11.0; Leica SP5 II = 1.7, 2.9, 5.7, Fig. 8d). These data demonstrate the benefits of the simple excitation and collection light paths and the purpose selected hardware on TIMAHC.

**Figure 9:**
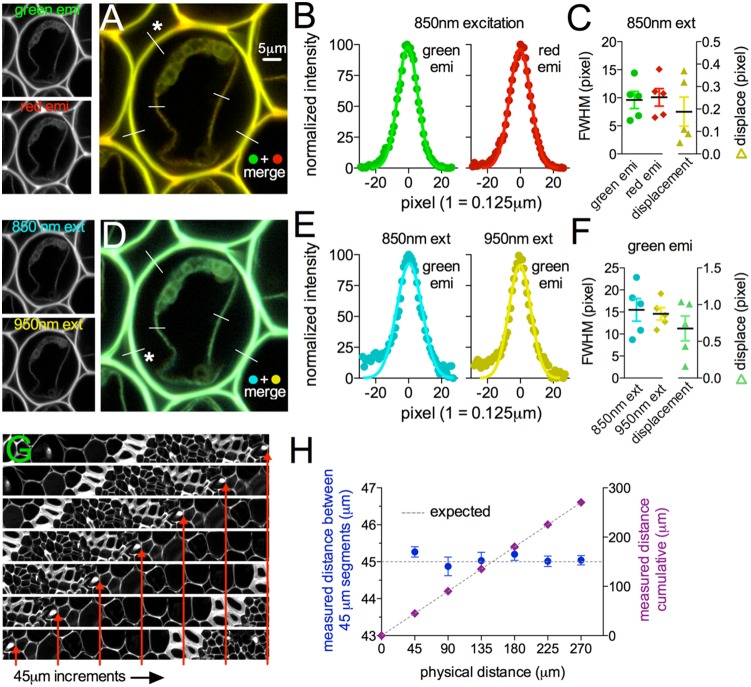
Microscope Performance: Tests for chromatic aberration and radial distortion. **A**) *Convallaria* sample excited at 850 nm collecting green and red fluorescence (small images left) (channels merged right). Lines represent profiles analyzed. **B**) Green and red structure width (FWHM) and location of peak (Gaussian fit), from line indicated with star in *A*. **C**) Summary showing the pixel distance of the green and red FWHM and the pixel distance difference between the two curve fit peaks. **D**) *Convallaria* sample excited at 850 nm and 950 nm collecting green fluorescence (small images left) (images merged right). Lines represent profiles analyzed. **E**) 850 and 950 structure width (FWHM) and location of peak (Gaussian fit), from line indicated with star in *D*. **F**) Summary showing the pixel distance of the 850 and 950 FWHM and the pixel distance difference between the two curve fit peaks. **G**) Sequential images at the full field of view (cropped rectangular), each taken after the stage was moved 45 microns in one linear direction by the motorization. Red arrows point to a particular feature to show the translation across images. **H**) Expected (dotted line) and measured (symbol) distances of a fine feature as it was moved at 45 micron increments across the full field of view. Segmental (blue circle) and cumulative (purple diamond) measurements were made.

To confirm that TIMAHC did not suffer from chromatic aberration or radial distortion, we imaged a uniform fluorescence standard with fine structures that could be broadly excited and was broadly emitting (*Convallaria*). To test for chromatic aberration, we quantified the fluorescence signal overlay using supra-Nyquist pixel sampling (pixel = 0.125 µm) between 1) green emission and red emission when excited at a fixed wavelength (850 nm), and 2) the signal overlay between the green emission when excited at two different wavelengths (850 and 950 nm). The difference in the FWHM of fine structures (green vs red emission: 0.46+/−0.08 pixel or 0.057+/−0.01 µm, n = 5, Fig. 9a–c; 850 vs 950 excitation: 2.55+/−0.3 pixel or 0.31+/−0.03 µm, n = 5, Fig. 9d–f) and the difference in xy coordinates of the peak of these structures (green vs red emission: 0.18+/−0.06 pixel or 0.057 µm, n = 5, Fig. 9a–c; 850 vs 950 excitation: 0.90+/−0.28 pixel or 0.11+/−0.03 µm, n = 5, Fig. 9d–f) was below the resolution limit. To test for radial distortion, we moved a fine structure (cell wall) on the *Convallaria* sample across the full field of view at regular physical distances (45 microns, as defined by the calibrated motorization) and took single images at the different distances travelled. We found that linear distance measurements of the expected 45 micron steps were accurate to 45 microns within the resolution limit (expected = 45 µm; measured = 45.07+/−0.05 µm, n = 3, Fig. 9g, h) and that the cumulative measurements accurately tallied within 0.5 microns to the total expected distance (expected = 270 µm; measured = 270.43+/−0.25 µm, n = 3, Fig. 9g, h). These data suggest that the custom optical train on TIMAHC is not impaired by chromatic aberration or radial distortion.

### Microscope Biological Sample Performance

#### Ethics Statement

The work was performed in strict accordance with the recommendations from the animal care and use committee of the University of Calgary. All procedures were approved by this committee and detailed on institutional protocols M11002 and M11032. All efforts were made to eliminate or minimize suffering. Surgical procedures used isoflurane anesthesia at the level of reflex suppression (5% induction, ∼2% maintenance) and post surgery buprenorphine analgesic was used (IP 0.1 mg/kg). For endpoints, animals were deeply anaesthetized using isoflurane and decapitated using a rodent guillotine to either prepare tissue for the acute brain slice experiments or after *in* *vivo* imaging finalized.

**Figure 10:**
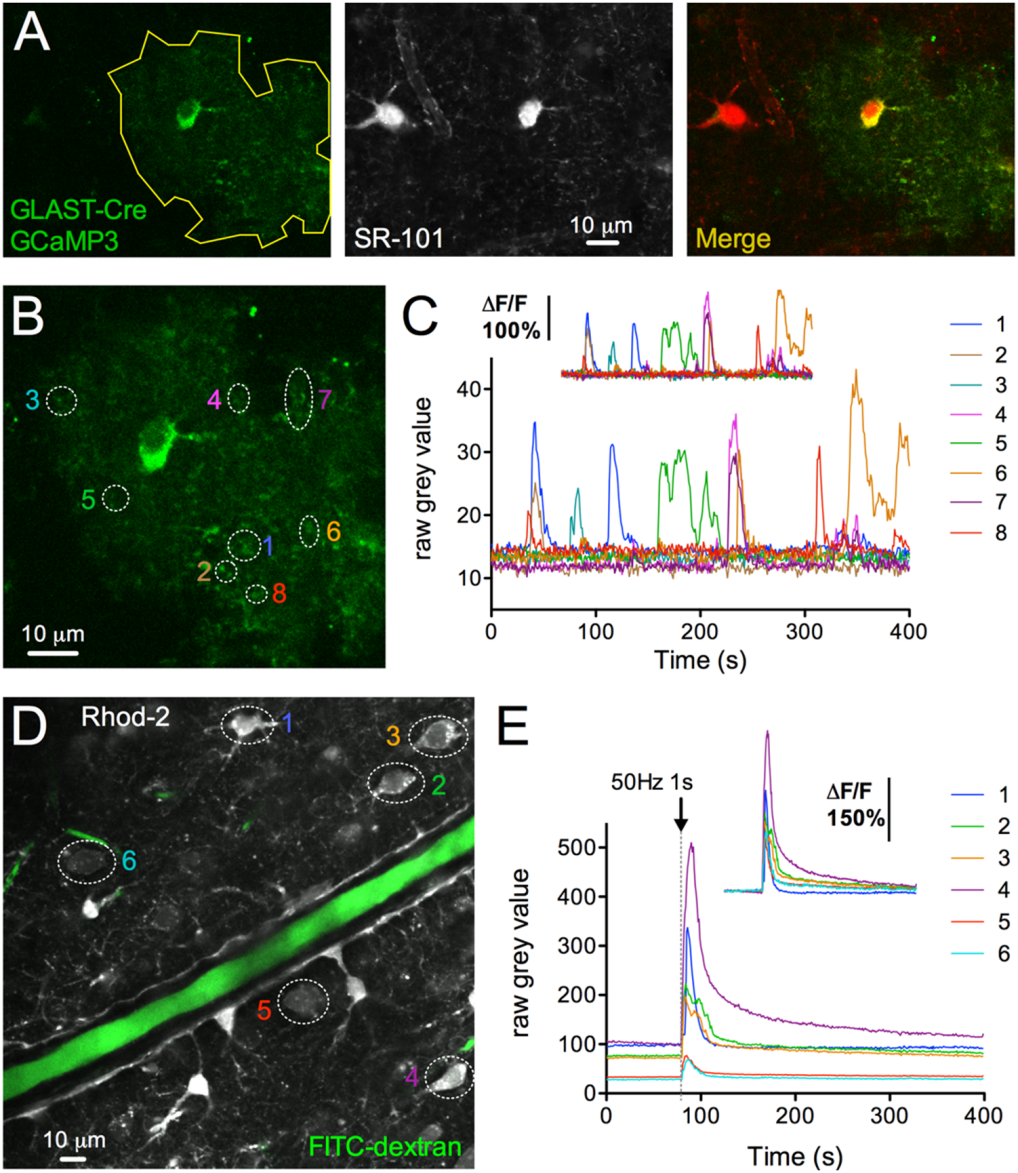
Microscope Performance: Ca2+ imaging in acutely isolated tissue. **A**) Two-photon fluorescence images of brain slices taken from GLAST-Cre LSL-GCaMP3 mice. The expression of the Ca2+ indicator GCaMP3 in astrocytes is shown by colocalization to SR-101. Astrocyte arbor outlined in yellow. **B**) Astrocyte close up showing micro-domain regions of interest. **C**) Raw Ca2+ signals from the regions of interest in *B*. Insert shows the ΔF/F. **D**) Neurons and astrocytes loaded with Rhod-2/AM (grey) and an arteriole filled with FITC-dextran (green) in the neocortex. Regions of interest shown. **E**) Raw Ca2+ signals detected from the regions of interest in *D,* in response to 1s 50 Hz electrical stimulation of afferent fibers. Insert shows the ΔF/F.

We examined the ability of TIMAHC to detect faint, or bright physiological signals from acutely isolated tissue. Acutely isolated brain slices (prepared as previously described [28]) were made from Cre-Lox transgenic mice in which the astrocyte specific promoter GLAST (JAX #012586) caused cre-dependent expression of the genetically encoded Ca2+ indicator GCaMP3 (JAX #014538) (Fig. 10a, b). Due to use of hemizygotic GCaMP3 mice and a low GCaMP3 signal at resting levels of Ca2+ [29], we observed weak basal fluorescence in astrocytes when imaged at 940 nm (grey values 13.3+/−0.5) in comparison to SR-101 (grey values 137.5+/−0.8). Nevertheless, TIMAHC captured spontaneous micro-domain Ca2+ transients in astrocyte fine processes with excellent signal-to-noise (ΔF/F = 142.1+/−16.6, baseline SD = 1.2, n = 8, Fig. 10b, c). To examine a bright signal, we electrically evoked Ca2+ transients in astrocytes and neurons using a concentric bipolar stimulating electrode (FHC) and Grass S88X stimulator. This was performed in brain slices taken from Sprague Dawley rats (p25). Slices were bulk loaded with Rhod-2/AM (10 µM, 0.1% DMSO, 0.05% Pluronic acid, 45 min incubation at 34C). Rhod-2 is a synthetic Ca2+ indicator with a larger dynamic range, brighter resting signal, but with a similar Kd compared to GCaMP3 [29]. We excited Rhod-2 at 850 nm and found that brief electrical stimulation (1s 50 Hz at 1.6 V) of afferent axons produced large Ca2+ transients in neocortical neurons and astrocytes with little baseline noise (ΔF/F = 213.4+/−39.5, baseline SD = 0.5, n = 6, Fig. 10d, e).

**Figure 11:**
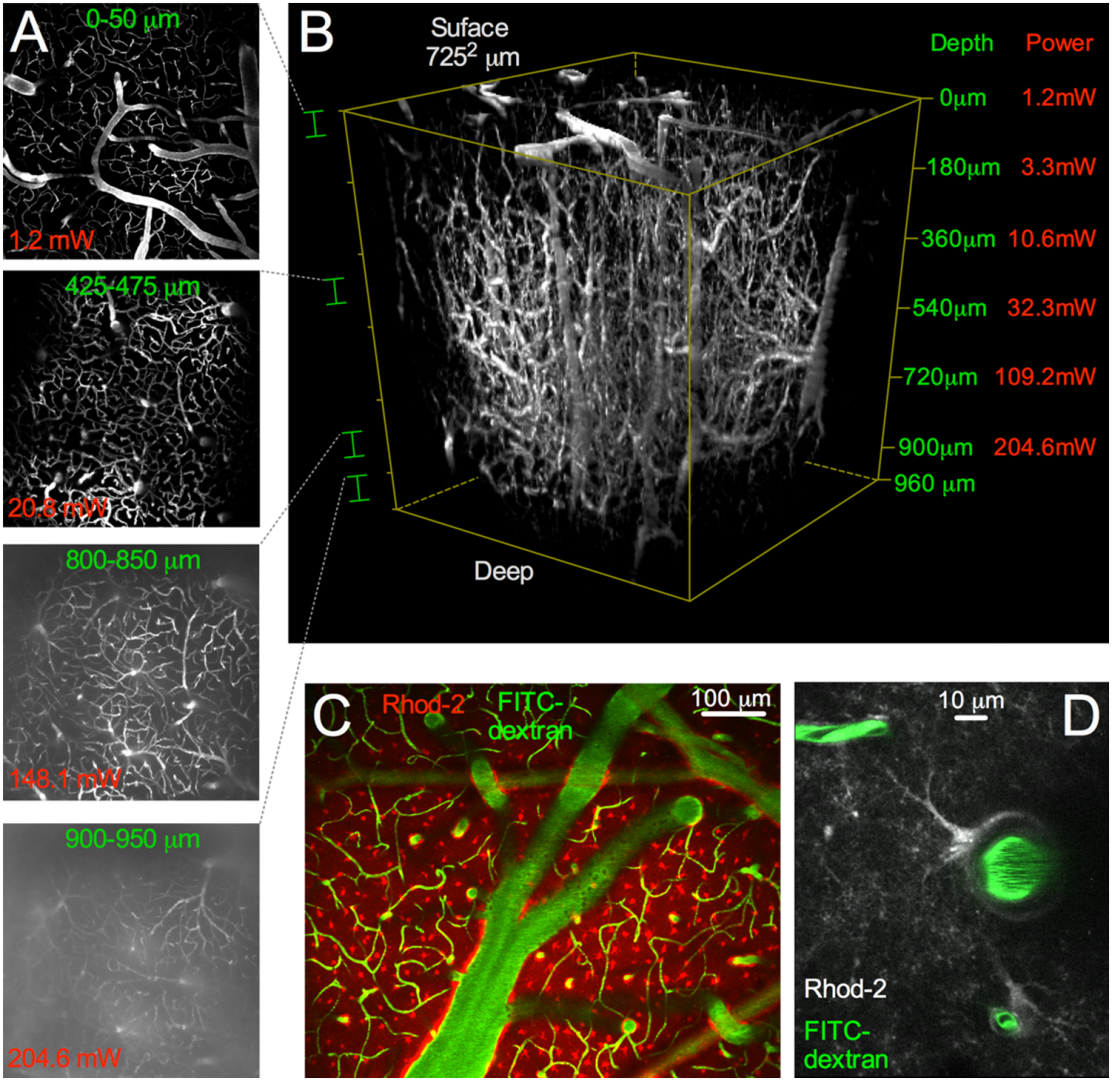
Microscope Performance: *In* *vivo* depth vs power and wide-to-small field Ca2+ imaging. **A**) Image depth versus power demonstration. Microvasculature filled with FITC-dextran. Four 50 µm thick max intensity stack images taken at different depths in mouse neocortex using a Nikon 16X 0.8NA objective lens, from the surface of the brain down to 950 µm. **B**) Complete 3D volume stack with a maximum depth of 960 µm (with background subtraction). Left side shows the sections of the stack that are shown in *A*. Right side shows the imaging depth versus the average excitation power at 780 nm. **C**) Wide field Ca2+ imaging with the Nikon 16X NA0.8 lens. Astrocytes were bulk loaded with Rhod-2/AM. **D**) Close field Ca2+ imaging. Single penetrating arteriole, capillaries (green), astrocyte endfoot and processes (grey) using the Zeiss 40X NA1.0 objective lens is shown.

To test how deep TIMAHC could image into highly light-scattering tissue, we performed a depth versus excitation power analysis in mouse cortex *in* *vivo*. For a uniform fluorophore concentration throughout the depth of the tissue, we tail vein injected FITC-dextran (7 mg in 0.15 mL lactated ringers) in a p35 C57Bl/6 mouse with an implanted cranial window [30]. Using a Nikon 16X 0.8NA objective lens, TIMAHC captured an ∼725^2^ µm view of the cortical microvascular network (Fig. 11a, b). At the surface of the brain, the average power of excitation was 1.2 mW at 780 nm. At depth, TIMAHC imaged up to 960 µm at an average excitation power of 204.6 mW at 780 nm until the background signal nearly equaled the signal coming from microvasculature (capillary grey value: 75.89+/−0.74, background grey value: 65.33+/−1.0, ratio 1.16, n = 5). At shallower depths, TIMAHC also imaged cell Ca2+ (astrocytes loaded with Rhod-2/AM) across a large field of view within the cranial window (Fig. 11c). Using the Zeiss 40X NA1.0 lens, fine cellular structures such as small penetrating arterioles, capillaries and astrocyte processes could be clearly visualized over a narrow field of view *in* *vivo* (Fig. 11d).

**Figure 12:**
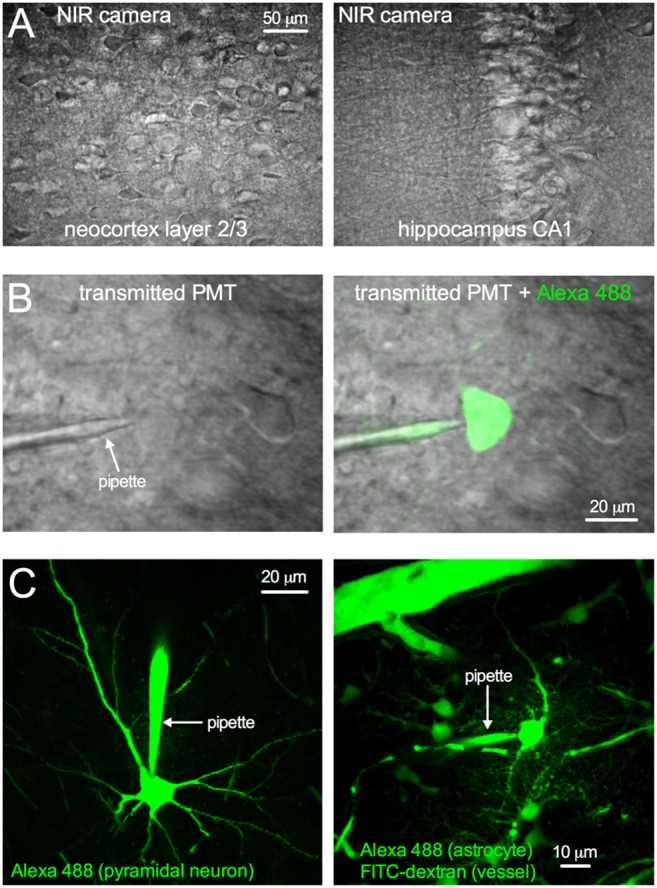
Microscope Performance: visually guided patch clamp. **A**) NIR LED and camera transmitted image of acutely isolated brain slices of the neocortex (left) and the hippocampus (right). **B**) Transmitted image of the tissue generated by the Ti:Sapph beam captured on the under-stage PMT. Patch pipette and cells can be visualized (left). A successful patch is shown with Alexa 488 fill (right). **C**) Whole-cell patch clamp of cortical pyramidal neuron (left) and astrocyte (right), each dialyzed with a standard K-Gluconate internal solution containing 100 µM Alexa-488 (Green). Images are displayed as a max projection to capture the cell(s) and the patch pipette.

Next we tested TIMAHC’s use for visually guided patch clamp. Either the LED and camera system was used for incoherent NIR patching (Fig. 12a) or cells were targeted for two-photon guided patch clamp [31] (Fig. 12b). We used a standard K-Gluconate internal solution containing 100 µM Alexa 488 sodium hydrazide (Life Technologies, A-10436) using methods previously described [28]. The under-stage PMT, which collects the transmitted signal from the tissue, was used to facilitate two-photon guided patching by allowing visualization of the patch pipette and the target cell itself (Fig. 12b). This permits patch clamp of an unlabeled cell or a fluorescing cell, without the need of a fluorescent molecule in the patch pipette (but shown here with dye to demonstrate the successful patch; Fig. 12b right). Once whole-cell, single cell recordings and fluorescence studies can be performed (Fig. 12c).

**Figure 13:**
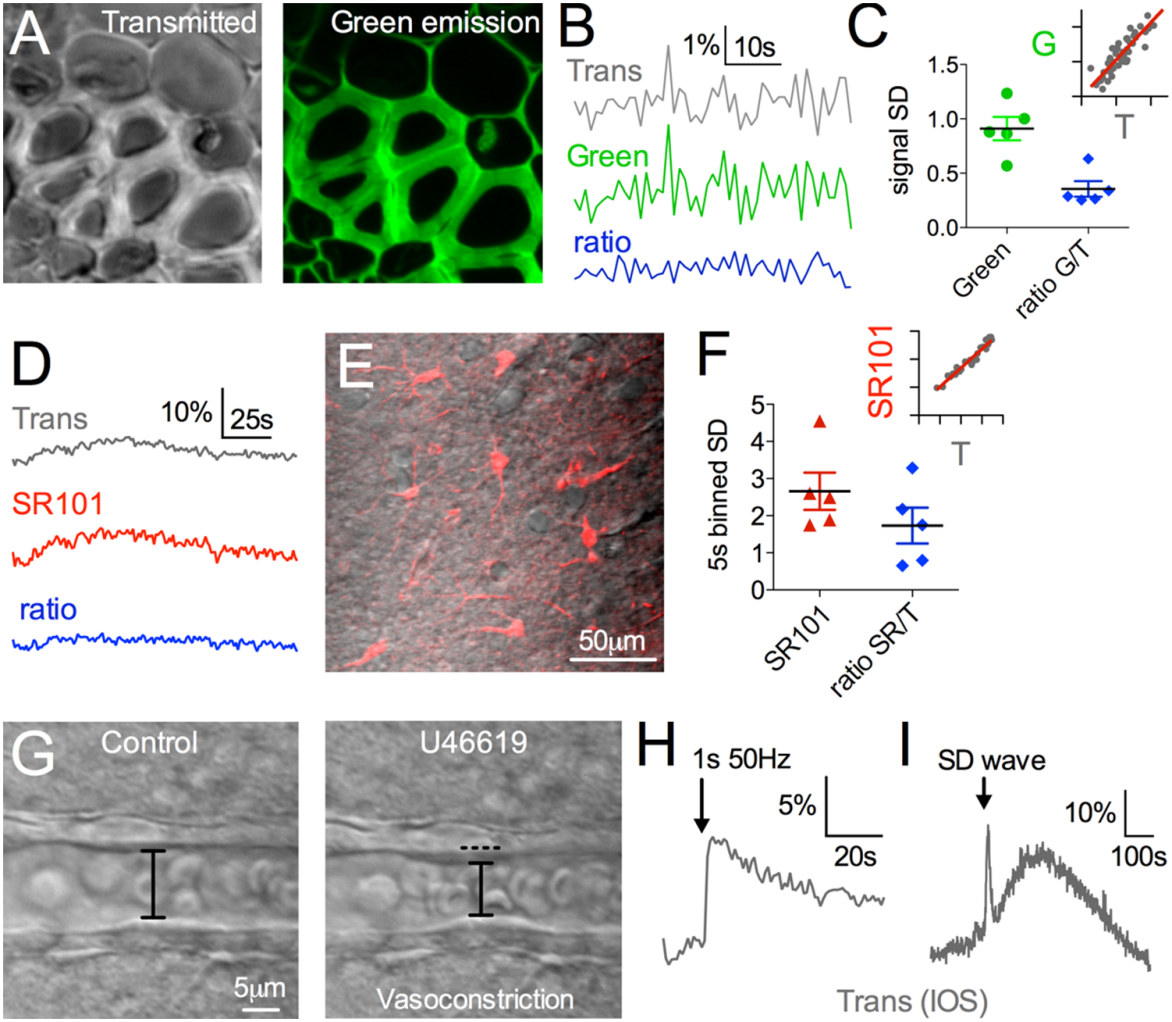
Microscope Performance: Under-stage transmitted channel. **A**) Transmitted image (left) and green fluorescence (right) of *Convallaria* standard sample. **B**) Traces showing fast correlated noise between the green and transmitted channel. The ratio of the two signals eliminates the synchronous noise and reduces the SD in the signal. **C**) Summary bar graph. Inset shows correlation between the green and transmitted signal r = 0.89. **D**) Traces showing slow correlated oscillations between the red (SR-101) and transmitted channel in isolated tissue, acquired from region shown in **E**. The ratio of SR-101/Trans largely removes the undulation and reduces the SD on 5 s binned data, summarized in **F**. Inset shows correlation between the red and transmitted signal on 5 s binned data (r = 0.97). **G**) Transmitted images of a small arteriole before (left) and after vasoconstriction (right) caused by the thromboxane receptor agonist U46619 (200 nM). Intrinsic Optical Signals (IOSs) captured by the under-stage PMT in response to synaptic activity (**H**) and a wave of spreading depression (SD) (**I**).

The under-stage PMT offered several other advantages during standard fluorescence signal capture. First, small synchronous signal fluctuations were made evident when we compared the fluorescence and transmitted collection (correlation r = 0.89, green emission SD: 0.91+/−0.10, transmitted SD: 0.85+/−0.17, n = 5, Fig. 13a–c). This correlated noise was not the result of electronic noise because the signals generated from a 1.5 V battery feeding each channel showed no correlation (r = −0.015+/−0.36, n = 3) and close to zero SD, and was thus likely the result of small fluctuations in excitation power. Notably, taking the ratio of the green fluorescence and the transmitted signal could dramatically lessen baseline noise (ratio Green/Trans: 0.35+/−0.07, n = 5, p = 0.002 compared to green fluorescence SD, Fig. 13c). Second, in isolated tissue over a longer time frame, we noticed another correlation made apparent by small undulations occurring in both fluorescence (SR-101 loaded astrocytes) and transmittance (Fig. 13d, e). To examine this slower relationship, we binned the data at 5 s to remove the synchronous noise seen at 1 Hz above (Fig. 13a–c). After binning, the two channels were still highly correlated (r = 0.86+/−0.06, n = 5, Fig. 13f insert) and taking the ratio reduced the undulations in the signal (SR-101 SD = 2.65+/−0.5, ratio SR-101/Trans SD = 1.73+/−0.5, p = 0.004, Fig. 13f). These data indicate that the transmitted channel can be used to detect small optical changes to the tissue that may influence fluorescence. This could aid an experimenter in understanding whether a small change in, for example, a Ca2+ indicator is the result of a change in Ca2+ or the result of a change in the optical properties of the tissue. Third, by visualizing the tissue via transmittance, one can capture gross structures, cell morphology/area, or as presented here, changes in blood vessel diameter (Fig. 13g). Finally, the under-stage PMT on TIMAHC can be used to study intrinsic optical signals from tissue, such as that generated by increases in synaptic activity [32] (Fig. 13h) or from pathological events such as spreading depression [33] (Fig. 13i).

### Alternative builds

TIMAHC can be readily adapted, expanded or simplified. We provide four additional models to serve as example modifications to TIMAHC: 1) a build with expanded table optics and kinematic sub-assembly so that TIMAHC can accept two laser beams; 2) a reduced build for *in* *vivo* investigations which possesses no NIR camera or under-stage optics; 3) the incorporation of a rapid shutter into the detector sub-assembly to block the PMTs during combined two-photon imaging and visible light photo-stimulation experiments when a fluorophore has an overlapping emission spectra with the excitation light and 4) a microscope build we call IMEPS, which stands for Infrared Microscope for Electrophysiology and Photo-Stimulation. IMEPS is not meant for two-photon fluorescence imaging and thus does not need to be coupled to a costly Ti:Sapph laser. However, one can collect the transmitted image of the tissue using a simple NIR diode laser (Thorlabs, part# CPS192), the galvanometric scanners and the under-stage PMT. The galvanometric scanners can also be used to select regions for photo-activation with a visible laser line that would be controlled by a shutter and/or Pockel’s cell. Furthermore, by replacing the above-stage PMTs with LEDs, point photo-stimulation at the center of the field of view at various illumination dot sizes would be possible. Note, these alternative builds have not been fully tested and detailed parts and price lists are not provided (Model S2).

### Image and statistical analysis

All image analysis was performed using the open-source processing program ImageJ. Statistical analysis, which included t-tests (paired and unpaired as appropriate), linear regressions, and correlations were performed using Graphpad Prism 5 software.

### Caution

TIMAHC is presented with an exposed beam path to better show the build, but the light path should be covered to increase safety using lens tubes and plastic tubes (Thorlabs, parts# SM1L*XX,* SM2L*XX* and SC1L24, SC2L24). Furthermore, though this resource is intended to facilitate the non-expert user in building a two-photon imaging system, it must be stressed that such an endeavour is a significant undertaking. It is the responsibility of the user to test, troubleshoot and fix. While we have tried to provide solid guidance in the building and troubleshooting sections in the supplementary information (Appendix S1), our effort on this front is not exhaustive.

## Discussion

In our laboratory TIMAHC has proven to be a robust research tool at an affordable cost. TIMAHC: 1) performs close to the theoretical diffraction limit, 2) has a signal-to-noise ratio that exceeds three commercial two-photon microscopes, 3) can image at depths nearing 1 mm in mouse brain, 4) is not impaired by chromatic and spherical aberration or radial distortion, 5) is capable of wide field imaging using low magnification but high NA objective lenses, 6) captures robust Ca2+ signals from cellular and subcellular compartments and 7) can be readily adapted.

One of the most important performance attributes of a fluorescence microscope is the signal-to-noise ratio. We tested this at three different excitation powers while keeping a constant max pixel grey value to permit contrasting between different imaging systems. TIMAHC displayed excellent signal-to-noise, however, in the comparison presented, it is important to clarify that there were variables that could not be kept constant between the different two-photon systems such as the objective lens model, the PMT model (though the Nikon A1 also possessed GaAsP PMTs), several optics and the complexity of the excitation and collection light path. The face-value comparison is valuable though, as equating all or most of the variables between the systems nullifies the test. It was the overall design and the parts chosen for TIMAHC that was of interest to compare to out-of-the-box commercial systems to gauge performance differences.

Despite the custom optical axis on TIMAHC composed of a generic scan lens and tube lens that feed into different commercial objective lenses, we measured the resolution close to the theoretical limit dictated by the diffraction of light. We also found that the system did not have detectable chromatic and spherical aberration, or radial distortions such pincushion or barrel warping of the field of view. This was partly expected because much of the microscopes imaging performance hails from the NA of the objective lens and the corrections made within objective lens. Additionally, the scan lens corrects for the off-axis light generated by the scanning mirrors to prevent warping of the plane of focus. The scan lens chosen for TIMAHC was designed for implantation into custom imaging setups (Thorlabs, part# LSM04-BB). Commercial tube lenses typically optimize parfocality (to keep the same image plane) when coupled to their commercial objectives (see Considerations and Limitations section). We chose a long focal length tube lens to minimize system artifacts caused by off-axis rays, and because longer focal lengths increase flexibility for adding optics into the light path.

TIMAHC imaged an impressive 960 µm deep into mouse cortex (p35). This tissue type and age has a light scattering length constant of approximately 100 µm [16] and the image depth achieved is close to the theoretical limit at this wavelength (780 nm) [15]. Thus, even deeper imaging should be possible using longer wavelengths to image red fluorescence molecules, as long as excitation power is not limiting [14]. The excellent signal-to-noise, the geometry of the collection light path and the use of a low magnification yet high NA objective lens likely contributed to the imaging depth of close to 1 mm [16].

The design foundation of TIMAHC, in which different sub-assemblies slide onto a main optical rail, provides great flexibility on the ultimate height of the objective lens. For instance, for *in* *vivo* imaging, it is only the relative position between the upper kinematic, scanning and detector sub-assemblies that is critical, not their absolute height off the optical table. All three sub-assemblies can be slid up or down the rail together to change the z space allocation under the objective for different experimental preparations. Furthermore, the condenser sub-assembly can be easily removed from the rail, and the sample stage can either be height adjusted, or aspects removed. Therefore, nearly any research preparation could be imaged in a relatively short time frame, such as rabbits, cats or even monkeys [34]. Even an awake mouse preparation, in which the animal performs treadmill running [35], requires a great deal of space under the objective lens. We are not aware of any commercial two-photon microscope that can make such dramatic alterations in geometry, while also being able to regain *in* *vitro* sample imaging using under-stage optics, with relative ease.

We provide four additional 3D CAD models detailing likely adaptations to TIMAHC, but many other modifications may be possible. First, TIMAHC could be adapted for second harmonic generation imaging. In this scenario, we recommend reconfiguring the table optics sub-assembly and the condenser sub-assembly following the parts and protocols described elsewhere [36]. Second, Ti:Sapph lasers can either include or be supplemented with an optical parametric oscillator for longer wavelengths and thus deeper tissue imaging [14]. While TIMAHC could perform longer wavelength imaging, its electro-optical modulator and optics are optimized for a Ti:Sapph output between 700–1100 nm. Thus, if wavelengths beyond this range were going to be the primary approach, we recommend choosing a more appropriate Pockel’s cell (such as the LTA crystal series from Conoptics), as well as mirrors and lenses with coatings that would optimize reflectivity and transmission (silver mirrors or Thorlabs C coating). One should also ensure the objective lens of interest has been tested for good transmission in the extended NIR wavelength range. Third, TIMAHC can be adapted for imaging with ultra-short laser pulses, so that a broad spectral bandwidth could be used to excite multiple fluorophores simultaneously. As ultra-short pulses are highly prone to dispersion, dispersion compensation mirrors (Thorlabs, part# DCMP175) and mounting hardware should be purchased, and perhaps instrumentation for measuring the pulse width.

Being comprised of opto-mechanical parts and possessing an open architecture, TIMAHC can be used as a teaching tool for advanced microscopy. For example, a few students working with an instructor could build TIMAHC as a semester long course. Combining building, testing, measurement and troubleshooting with topic lectures could teach many different principles about the microscope. This includes but is not limited to: basic optics, light sources, the optical path, scanning, objective lenses, detection and many other details about the imaging modalities. This would be experiential learning at its highest level.

## Considerations and Limitations

There exist a number of discussion points that any prospective individual must consider before using this resource. First, TIMAHC incorporates 5 mm scanning mirrors from Cambridge Tech and given the beam expansion provided by scan lens and tube lens (3.7x) the max beam diameter at the back aperture of the objective lens is 18.5 mm. Thus, TIMAHC is optimized for the use of objective lenses with back apertures less than this diameter. If larger or smaller max beam diameters are desired or if different max scanning speeds are needed, a few items can be changed/modified in the scanning sub-assembly. First, use of larger scanning mirrors will permit larger beam diameters but will slow down the max scanning speed. Conversely, smaller mirrors reduce the max beam diameter but allow faster scanning. Furthermore, the two custom machined pieces in TIMAHC’s scanning sub-assembly will need modification to fit a different scanning mirror mount if the scanning mirrors are changed. Changing the focal length(s) and thus the expansion ratio of the scan lens and/or tube lens can also modify the beam diameter. Note that the distance relationships between the scanning mirrors, scan lens, tube lens and objective back aperture will need to change to suit the new focal length(s) [7], [8] and thus the distances in the provided supplementary model will no longer apply.

Alternatively, one can keep TIMAHC’s configuration and be aware of the shortcomings of back apertures that exceed 18.5 mm. First, the objective will not have diffraction-limited performance [1], [8]. This is acceptable if the larger field of view is of interest, rather than the sub-micron scale. For example, we use the Nikon 16X 0.8NA lens (back aperture 20 mm) routinely to map brain micro-vasculature and localize spatially distributed Ca2+ signals *in* *vivo* using sub-Nyquist pixel sampling (1024^2^). In fact, Nyquist sampling with a diffraction limited high NA, low magnification lens requires a very high pixel density and such a slow frame acquisition that this approach can be impractical, especially with galvanometric scanners. Second, large back aperture objectives may result in a small signal loss as we use a 1 inch collection lens as the first lens in the detector sub-assembly after the primary dichroic. This is because emitted fluorescence from scattered, non-ballistic photons emerging from a large back aperture may miss the 1 inch collector lens [1], [24]. However, TIMAHC’s fixed, short-length collection path is intended to minimize signal loss. The imaging depth obtained (960 µm) suggests the 1 inch collector lens is not a major limitation. Finally, the parfocal length between different objective lenses (such as between high NA objectives with drastically different magnifications) can be so significant that the 1 inch range on the z slider cannot accommodate the use of both lenses at a given height of the sample stage. Either the sample stage and condenser height can be configured and optimized for a particular objective, or one can add extension collars (Thorlabs, part# SM1L*XX*) to the shorter objectives to equalize the parfocal distance.

For TIMAHC’s scanning hardware we recommend specific maximum bi-directional scanning speeds at particular zoom factors (or rather particular scan angles) to not exceed the capabilities of the system. By testing sustained scanning stability at various speeds, we found that stable imaging is achieved when not exceeding these values: zoom 1 at 2 ms/line, zoom 1.3 at 1 ms/line, zoom 3.5 at 0.5 ms/line, zoom 10 at 0.25 ms/line (data not shown). All of these settings keep the amperage drawn from the servo scanning board to less than 0.75 Amps per channel, which avoids aberrant scanning behavior.

We use a generic, long focal length (200 mm) achromatic tube lens (Thorlabs, part# AC508-200-B). When using a commercial objective lens that is not designed for the tube lens, the transmitted image generated by the under-stage LED will not be parfocal with the two-photon generated image. When using the 40X 1.0NA water dipping objective from Zeiss we find that these two image planes are offset from each other by approximately 150 microns. This is easily overcome by moving the objective lens this z-distance when switching from the LED transmitted light imaging to the two-photon imaging or vice versa. Importantly, a generic tube lens is used on TIMAHC so that we can choose the best commercial objective available for a particular experimental need and do not have to stay loyal to a specific brand.

The number of fluorescence detectors and associated optics/hardware is expandable but the weight imposed on the z motorization slider needs to be considered. For more fluorescence channels a different design is recommended, in which the detector sub-assembly is fixed to the main optical rail (while maintaining the optical axis) and not directly to the z motorization. If adding more fluorescence detectors in series to the assembly, the focal lengths of the lenses in the detector sub-assembly will have to change in order to focus the emitted fluorescence properly to each PMT [7].

In using this resource one must consider the cost versus time tradeoff. Though the cost savings on TIMAHC can easily be in excess of $150 K USD for a comparable two-photon microscope, considerable time is required for building and in some cases troubleshooting. For instance, one to two experienced people (directly or technically inclined) would take two to three weeks to achieve an optimized system. With little experience, one to two people may take a couple months. Furthermore, it is difficult to predict what troubleshooting will be needed, which could protract the process. We have provided as many potential problems and solutions as possible in the supplemental material to speed the finalization process (Appendix S1). The lead-time on certain parts for TIMAHC also needs to be considered. The Ti:Sapph laser, the fluorescence optics and the GaAsP PMTs have the longest lead-time (up to a few months) yet this is comparable to the lead-time on complete commercial systems.

## Supporting Information

Figure S1 **Detector sub-assembly mounting alignment aid.** Close up of the detector sub-assembly (PMTs and objective lens removed) and the bottom aspect of the scanning sub-assembly. Parts coloured in blue (CP02, ER6 x 2, LCP02) are the mounting alignment aid. To add the alignment aid the tube lens should be removed from the scanning sub-assembly. The bottom of the CP02 plate should touch the top of the primary dichroic cube such that the back aperture of the objective lens will be approximately 200 mm from the middle of the tube lens. Importantly, this position is when the z slider has been descended almost to its full extent (the imaging position, not when the objective lens has ascended for tissue loading or pipette approach). The tool is necessary to mount the detector sub-assembly level on the optical axis and at a desired angle to create space for micromanipulator access to the tissue bath.
(TIF)

Table S1 **A complete parts and price list for TIMAHC.**
(XLSX)

Appendix S1 **A guide to assist in building, testing and troubleshooting TIMAHC.**
(DOCX)

Model S1 **A 3D CAD model of the complete microscope is provided, including separate models for each of the sub-assemblies that comprise the full assembly.** The exact position and orientation of every part within the build can be visualized. Models are viewed in SolidWorks eDrawings (a freely available software for online download) or SolidWorks proper.
(ZIP)

Model S2 **Four alternative builds or modifications to TIMAHC are provided as 3D CAD models.** These include 1) a simplified model for only *in* *vivo* applications, 2) a model in which TIMAHC can accept two beams, such as for imaging and visible line stimulation experiments, 3) an alternate detector assembly with a rapid shutter to protect the PMTs during visible light photo-activation and 4) a microscope build call IMEPS that is not used for fluorescence imaging, but instead for Infrared Microscopy combined with Electrophysiology and Photo-Stimulation. Each alternative build contains the complete model, as well as separate models for each subassembly contained within the full build. Models are viewed in SolidWorks eDrawings (a freely available software for online download) or SolidWorks proper.
(ZIP)
