# Supplementary figures and images for: A High Performance, Cost-Effective, Open-Source Microscope for Scanning Two-Photon Microscopy that Is Modular and Readily Adaptable
Source: PLoS One. 2014 Oct 21;9(10):e110475. doi: 10.1371/journal.pone.0110475 (PMC4204885; doi:10.1371/journal.pone.0110475)

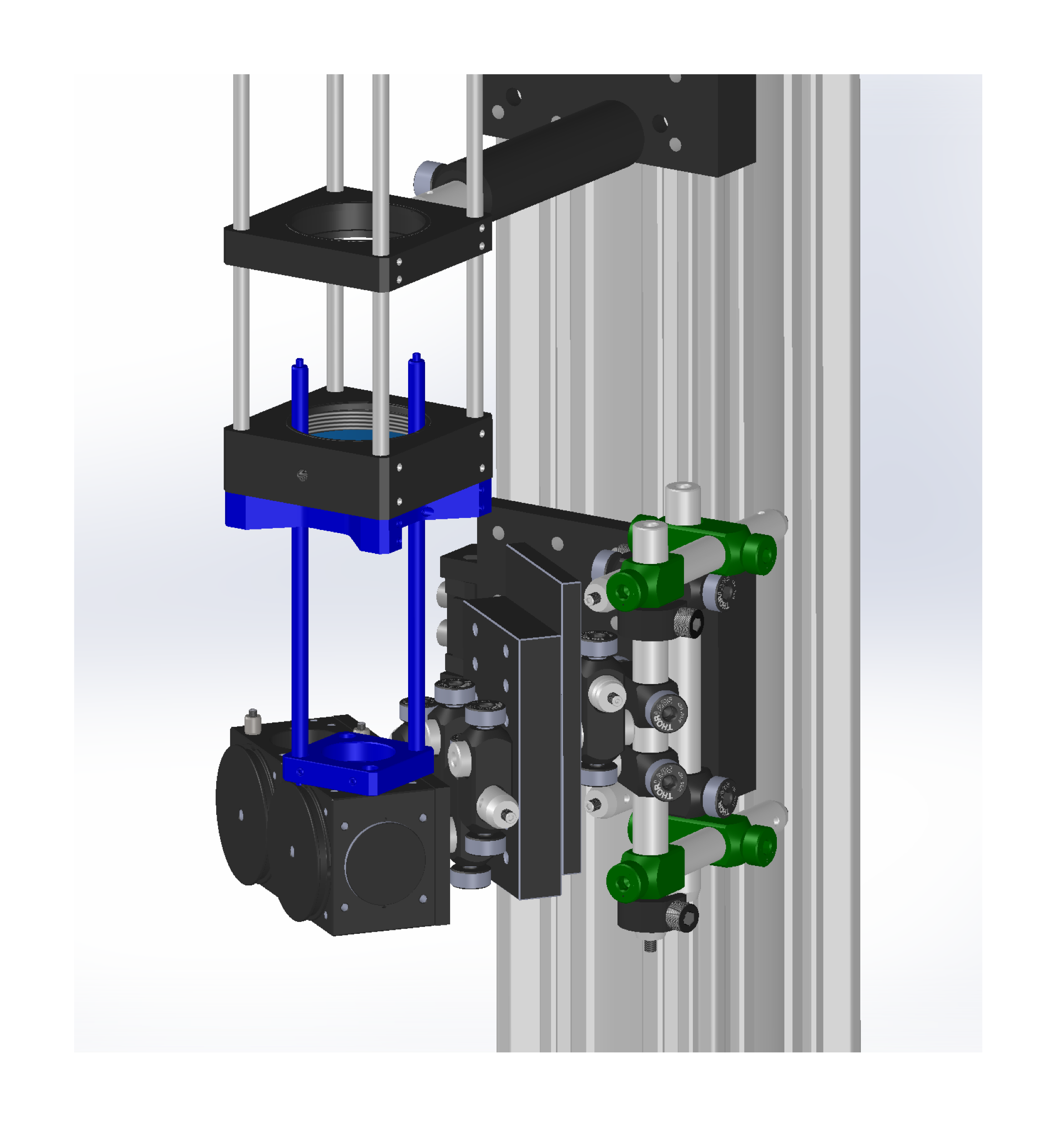

Supplement: Figure S1 — Detector sub-assembly mounting alignment aid. Close up of the detector sub-assembly (PMTs and objective lens removed) and the bottom aspect of the scanning sub-assembly. Parts coloured in blue (CP02, ER6 x 2, LCP02) are the mounting alignment aid. To add the alignment aid the tube lens should be removed from the scanning sub-assembly. The bottom of the CP02 plate should touch the top of the primary dichroic cube such that the back aperture of the objective lens will be approximately 200 mm from the middle of the tube lens. Importantly, this position is when the z slider has been descended almost to its full extent (the imaging position, not when the objective lens has ascended for tissue loading or pipette approach). The tool is necessary to mount the detector sub-assembly level on the optical axis and at a desired angle to create space for micromanipulator access to the tissue bath. (TIF) [file pone.0110475.s001.tif]
