# Supplementary material for: A High Performance, Cost-Effective, Open-Source Microscope for Scanning Two-Photon Microscopy that Is Modular and Readily Adaptable
Source: PLoS One. 2014 Oct 21;9(10):e110475. doi: 10.1371/journal.pone.0110475 (PMC4204885; doi:10.1371/journal.pone.0110475)
Supplement: Appendix S1 — A guide to assist in building, testing and troubleshooting TIMAHC. (DOCX) [file pone.0110475.s003.docx]

Appendix

General assembly: We recommend building each sub-assembly off the rail first before adding them. The sliding rail plates, to which most sub-assemblies are mounted, allow each of the sub-assemblies to be placed on or taken off the main rail independently and easily. Furthermore, the Solidworks models of the sub-assemblies and the full assembly are done as accurately as possible. If the builder is unsure about the exact position or distance of a particular element, accurate measurements can be taken directly on the model using the measurement tool either in Solidworks proper or Solidworks eDrawings. The latter is a free software for download. Finally, we do not provide part-by-part, step-by-step instructions on how to build TIMAHC. Instead the builder should examine each of the sub-assemblies carefully so that the building process becomes relatively self-explanatory. However, the order of operations may be important in some circumstances and when specific steps might not be obvious, we provide some helpful tips for building below.

Tips for building and mounting the scanning sub-assembly: The order of operations is important when building the scanning sub-assembly. It starts with the two base plates that attach to the main optical rail (Thorlabs part# XT95P11). One should build progressively towards the optical axis and pay close attention to when cage rod systems are nested within other cage rod systems. One should also ensure that the sliding cube (Thorlabs, part# C6W) is added before the vertical 2” cage system closes off the entry point, and also before any camera elements are added. The cage system that supports the sliding cube must be perfectly square so the cube slides effortlessly. If the cube does not slide nicely, discrepancies in the structure must be optimized. The angle of the mirror in the sliding cube may need adjustment to be centered to the camera (Dage MTI, part# IR-1000). This can only be done once the condenser assembly is setup because a transmitted image is needed for the alignment. Next, one should be very carful when mounting the scanning mirrors and do not touch the mirrors themselves.

Tips for building and mounting the detector sub-assembly: When inserting the collector lens (Thorlabs, part# LA1708-A) that rests between the primary and secondary dichroic cubes (Thorlabs, parts# C4W and C6W), one must first thread a lens retaining ring into the appropriate 1" through-hole on the primary cube. Then, another retaining ring must be threaded into the apposed hole on the secondary cube. When the two cubes come together the distance between the two rings needs to perfectly sandwich the lens. If the distance between the two rings is too long, the lens will rattle. If the distance between the two rings is too short, the two cubes faces will not sit flush with one another. The distance between the retaining rings will likely need to be adjusted until the collector lens is held properly. Next, when the fluorescence optics are added into the detector sub-assembly, one must be sure to wear powder free gloves and to note the orientation of the optics. For the Chroma optics used here, Chroma's convention is to have an arrow point to the reflective side of the dichroic (695cxxr and T560LPXR). For emission filters (ET525/50m-2P and ET605/70m-2P), the arrow should point towards the light source or rather point in the opposite direction that the fluorescence light travels. Finish building the detector sub-assembly, with the attached z-slider (Sutter, part# MP-285-1z), up to the point where the RA180 parts (coloured green) and the horizontal TR3 posts are installed. Next, build the mounting platform until the two RA90 parts (coloured green) are installed and add this apparatus to the main optical rail at a height specified in the model. Then, suspend Thorlabs parts# CP02, 2 x ER6 and LCP02 from the cage hardware directly above in the scanning sub-assembly to serve as an optical axis mounting target/alignment aid (fig. S1). The bottom of the CP02 cage plate should touch the top of the primary dichroic cube (C4W) at a height such that the back aperture of the objective lens will be ~200 mm from the middle of the tube lens. The distance of ~200 mm is for when the z-slider/objective lens is in the imaging position (descended to nearly its full extent) rather than when the objective lens has been ascended/retracted for loading tissue or pipette approach. The tube lens, located at the bottom of the scanning subassembly, should be removed for mounting the alignment aid (fig. S1). Next, ensure that the green coloured RA180 and RA90 parts are loose (screw not tightened). Take the assembled detector and z-slider (!!PMTs and objective lens removed!!) in hand and place a calibrated circular bubble level (Edmund Optics, part# 39-435) on top of the secondary dichroic cube (C6W). Then slide the two free horizontal TR3 rods into the loose RA90 connectors (green) located on the mounting assembly. While holding the detector assembly with the green parts still loose, one must achieve three things: a) center the 1” through-hole on top of the primary dichroic cube with the through-hole of the CP02 plate; b) level the detector assembly using the circular bubble level or see if the top of the primary cube can contact perfectly flush with the bottom side of the CP02 plate; c) rotate the entire detector assembly (so that the lower PMT turns towards the main optical rail) to a desired angle to increase the amount of space at the front of the sample stage for manipulators. Once these three aspects are achieved, one can tighten the green components by hand and then double check the position. If it is satisfactory, one can tighten all the green parts with an Allen key. If it is not satisfactory, the green parts can be loosened and the steps can be repeated.

Tips for condenser setup and alignment: First, one should be sure to note the orientation of the 50/50 beam splitter cube (Thorlabs, part# CM1-BS015) at the bottom of the condenser sub-assembly. The light-path-lines drawn on the cube should connect all three paths to the LED, the PMT and the sample. Second, the builder should be aware that due to space constraints of the design, the field lens (Thorlabs part# AC254-30-B) rests passively in its lens mount, so one needs to avoid positions in which the lens may fall out of place during the building and/or installation process. Third, the height of the condenser sub-assembly relative the bottom of the tissue bath to achieve Koehler Illumination is not intuitively obvious. As a general guideline, the builder needs to raise the sliding base plate (Thorlabs, part # XT95P11) up the optical rail until the condenser lens (Thorlabs, part# C330TME-B) is as close to the bottom of the bath (the cover glass) as possible without touching it. From this position, adjustments on the fine z translator (Thorlabs, part# SM1Z) should be adequate to bring a closed field iris into focus. Additionally, with the field iris mostly closed, use the xy slip plate (located under the field lens) to center the condenser lens to achieve Koehler. Once the condenser assembly is in position, the distance of the collimating aspheric lens (Thorlabs part# ACL2520-B) directly in front of the 940 nm LED may need to be adjusted to optimize the collimation. We recommend looking at the overall brightness and uniformity of the transmitted light on the IR-1000 camera (though this will need to be setup and aligned well). The brightest image with the most uniform illumination is desired. Once this is achieved, this lens can be tightened into position.

Tips for wiring and setting up the scanning mirror system: The scanning mirror system from Cambridge Tech Inc. requires two custom cables, heat sinking and mounting of the servo driver board (673XX), as well as mounting the galvanometric scanners (6210H) on top of the scanning sub-assembly. Cambridge supplies the plastic connectors and metal pins needed for the cables, but the user must supply wire, BNC cables, banana plugs and perform the soldering (see Table S1, the main parts list). To make the cable(s) that send voltage signals from the command hardware (National Instruments breakout box BNC-2090A) to the scanning servo driver board, cut one 6ft BNC cable in half and separate out the core (signal wire) from the external mesh wire (ground) on both halves. These two cables will convey signals for the x and y mirrors. Next, 22 gauge stranded hookup wire (McMaster Carr, part# 8054T13) is soldered to the ends of each cable. The free ends of the hookup wire should then be exposed and soldered to the small female metal pins supplied by Cambridge. Following the Cambridge instructions, the four female metal pins are fed into a six slot (3 by 2) plastic connector in the proper configuration (!!see problem/solution #6 for this step!!).

To make the cable(s) that power the mirror board, we use 16 gauge (McMaster Carr part# 8054T16) and banana plugs (Digikey, part# J10140-ND). Four bare wire ends are soldered into the female pins and subsequently inserted into the plastic four pin connector supplied by Cambridge. One then solders banana plugs to the other ends of the wires. Two of the four wires are grounds and, as per the vendor diagram, we solder these ground wires together at some point between the plastic four pin connector and the banana plug ends. One then follows the vendor instructions for connecting the banana plugs to the power supply (Topward, part# 6303D).

Next, the servo driver board needs a heat sink and to be mounted. Tap three M3 holes on the solid plate of the heat sink (Digikey, part# ATS1359-ND) roughly in the center and spaced to match the holes in the servo driver board. Scrape a thin layer of standard computer thermal paste on the two surfaces that are to come together. Next, join the heat sink and the driver board, thread the M3 screws (Thorlabs, part# HW-KIT/M) and finger tighten. A jumper on the board may need to be removed to thread and tighten the center M3 screw. Return the jumper once finished. One needs to ensure there is solid and even contact between the board and the heat sink. The board then needs to be mounted on the middle backside of the main optical rail on the microscope. We take advantage of a through hole in the heat sink and use Thorlabs post hardware (see Table S1 main parts list) to mount the board on the back of the rail at about half height. A low vibration 80 mm computer fan can be mounted to the servo driver board heat sink to further dissipate heat if required.

Tips for wiring the GaAsP PMTs and pre-amps: Using the same stranded 22 gauge hookup wire and banana plugs as described above, connect the five colour-coded thin wires that emerge out of the back of a given PMT to the appropriate ports on the dedicated power supply (Hamamatsu, part# C7169). In addition to the thin cables, the single thick black wire emerging from the PMT is the signal cable. One needs to separate out the core (signal wire) from the external mesh wire (ground) on both this signal cable and a cut BNC cable (Hamamatsu, part# E1168-05). The signal wires from the PMT and the cut BNC are then soldered together and similarly the corresponding ground mesh wires are soldered together. Take care to insulate any exposed elements with electrical tape when finished. We also use a small zip tie to secure the signal cable to the BNC cable (joined cables make a hairpin turn) to relieve any stress on the solder joints. The free end of the BNC plugs into the input on the pre-amplifier (Sigmann Elektronik), which we fasten to the optical table. BNC outputs from the pre-amps then connect to the National Instruments hardware (BNC-2090A). The two pre-amps (one for each GaAsP PMT) are powered at +/- 12V (Topward, part #6303D). We use 18 gauge stranded wire (McMaster Carr part# 8054T15) and banana plugs to connect the voltage poles and ground between the power supply and the pre-amps.

Tips for wiring the under-stage PMT: Setting up the under-stage PMT is relatively straightforward. One must connect the signal output from the PMT to the data acquisition hardware using a SMA to BNC cable (Thorlabs, part# CA2812). The PMT comes with its own power supply, but the user must add a single channel low voltage power supply for gain control (Topward, part# 3185D). Finally, we find that grounding the PMT to the optical table helps reduce noise in the image.

Aligning the beam - Warning: Ti:Sapp lasers are very dangerous, especially to the eye, such that permanent vision loss can result from mishandling the Ti:Sapp beam. Laser safety goggles designed for a Ti:Sapp (Thorlabs, part # LG9) are necessary for beam alignment and are used in combination with anti-stokes beam illumination cards (Thorlabs, part # VRC5) so that the beam can be visualized in safety. Beam dumps are always used during the laser alignment process such that the beam is always terminated into a dump and never fires stray in an uncontrolled direction. The Gordon lab takes no responsibility for harm or injury that results from neglectful guidance of the Ti:Sapp beam. If your Ti:Sapp model has a non-mode locked, continuous wave setting with a reduced average power, this setting must be used for beam alignment. Reflective neutral density filters can also be used in combination with a beam dump to greatly reduce the power of the beam to improve the safety considerations. A laser safety course is also recommended before attempting to guide a Ti:Sapp beam into a microscope.

We aim to have the Ti:Sapp beam travel precisely along the tapped holes in the optical table and at a parallel height to the surface of the optical table. This is to ensure the beam will ultimately travel optimally down the optical axis of the microscope. Critical to this task is to achieve accurate 90-degree turns when the beam path necessitates a change in direction along the table as it travels to the microscope. Where a 90 degree turn in the beam path is needed, a post base plate (part# BE1) and clamp (part# CF125) is required (see table optics sub-assembly), rather than threading the post holder directly into the tapped holes of the optical table. This ensures that the face of the turning mirror can reflect the beam precisely along the center of the tapped holes in the optical table. To help position turning mirrors and guide the beam along the tapped holes, two additional posts and post holders that sport an alignment target (Thorlabs, part# SM1A7) are required. Both of these alignment aids are to be threaded directly into the optical table at different points along the path (for instance one before and one after a turning mirror). To ensure that the beam travels at a height parallel to the surface of the optical table, the center of the target must be exactly the same height as the beam leaving the laser head. The Pockel’s cell is added to its desired location in the path once the beam is in position. The precise axial position of the Pockel’s cell is controlled by an alignment mount following vendor instructions. Once in place and powered on, the Pockel's cell can be used to reduce the power of the beam (down to ~5 mW) for all subsequent downstream beam path work. At this point, neutral density filters may be removed. Using lens mount targets (part# LMR1AP) we ensure that the beam travels through the centers of the table achromatic lenses and that the height of the center of the lens matches the height of the beam above the table. Once the beam has entered TIMAHC, we use Thorlabs cage system targets (parts # CPA1 and LCPA1) and adjust the kinematic mirrors to direct the beam to the scanning mirrors and down through the optical axis. Fine adjustments on both the upper and lower kinematic mirrors are used to position the beam as best as possible through the center of the lenses (see problem/solution 1). Minor discrepancies of 1-3 millimeters from the lens centers are normal but the center of the objective lens must be hit as a final target. If the beam is more than a few millimeters off center from the scan lens and the tube lens then the path along the table must be scrutinized for any off tapped-hole or off 90-degree occurrences. Next, the beam must also be assessed for collimation from the tube lens down to the objective lens. Any divergence or convergence detected in the beam can be adjusted for using the translational slider (part# PT1) in the expansion and collimation lens pair on the optical table. Finally, the back aperture of the objective lens must be on a conjugate image plane to the scanning mirrors. The dimensions of the build are set to achieve this but it should be functionally checked. To do this, one needs to assess how much the beam moves back-and-forth at the approximate level of the objective’s back aperture when scanning. Both above and below the conjugate plane, the scanning-induced movement in the beam will be readily apparent, but at the conjugate plane the movement will be minimal. The level of the least movement in the beam should be the same level as the objective lens back aperture. If there is a discrepancy here, see problem/solution #10.

Testing and Troubleshooting: If using ScanImage software to control TIMAHC, one should follow the instructions on the Janelia open wiki website to configure the software and the data acquisition hardware. Once all of the building and setup is complete, including beam alignment, it is time to take your first image. To begin with, we typically image 1) a *Convallaria* sample, which is a broadly emitting, thin, uniform fluorescence sample (acquired from Zeiss from rep) from which the overall general quality of the image can be assessed or 2) sub-resolution Fluosphere beads (Life Technologies part# F-8803, 100 nm diameter) to quantify the microscope’s resolution limit and to identify potential optical problems in the point spread function [1]. If there are issues that go beyond subtle, sub-optimal optics, we use additional equipment such as oscilloscopes, signal generators and voltmeters to try and identify where in the microscope the problem is located. Below, we list some potential problems that can be encountered when first testing TIMAHC and some potential solutions.

1) Potential Problem: I cannot get the beam to travel down the optical axis well OR both of my fluorescence channels are bright on one side but dark on the other.

Potential Solution: First, the scanning mirrors may need to be powered and under software control, which specifies the starting position as well as any desired offset in the position. If using ScanImage, one needs to ensure the parked scanning position is 0.0x 0.0y and that there is no scanning offset either. Or trial different offset values to see if the optical axis can be achieved. Without performing these steps, the scanning mirrors might not be in the resting orthogonal position and instead directing the beam out of the optical axis of the microscope. Second, we describe how to use the lower and upper kinematic mirrors to optimize beam alignment down the optical axis. The lower kinematic mirror is used to place the beam at different spots on the upper mirror (not necessarily the exact center of the upper mirror). Then the upper mirror guides the beam into the scanning mirrors. One can start by adjusting the upper kinematic mirror. If the image cannot be made optimal, then the lower kinematic mirror can be adjusted. Here, we recommend using the lower kinematic mirror to systematically test different locations for where the beam falls on the upper mirror and then make adjustments to the upper mirror to try and optimize the image. For example, use the bottom mirror to position the beam onto the top-left quadrant of the upper mirror, then adjust the upper kinematic and examine the quality and uniformity of the fluorescence image. If not optimal, one can move the lower kinematic mirror so that it aims the beam onto the top-right quadrant of the upper mirror and repeat the steps. If you are not satisfied then one should test all quadrants of the upper mirror. One can also vary the distances the beam falls into a given quadrant on the upper mirror i.e. 2mm into the bottom-right quadrant from center, or 4mm into the bottom-right quadrant from center etc. Third, there could be discrepancies in how accurately the beam is traveling along the optical table. Double check the beam path that the tapped holes are followed, that precise 90 degree turns are made and that the height above the table remains parallel to the table surface. The last possibility that can explain both channels being equally compromised is that the primary dichroic mirror is not at the desired 45-degree angle.

2) Potential Problem: I can only see one fluorescence channel.

Potential Solution: First, test the computer hardware for the affected channel. Simply swap the BNC cable from the working channel’s PMT signal into the BNC port of the affected channel. If the functional channel still works you know the computer hardware is OK. If the functional channel stops working, the NI breakout box, the cable, and the data acquisition card should be tested to ensure they are functioning properly. You can start by using the scope control software (i.e. ScanImage) or the NI Max software to test if the affected channel can receive a signal. A common AA battery and two wires can be used to test if a 1.5V voltage signal can be detected by the system. Second, one needs to ensure that all the fluorescence optics are in the right location and proper orientation. For example, if the primary dichroic and secondary dichroic are in the wrong cube (e.g. primary is located where the secondary should be, and the secondary is where the primary should be) only green fluorescence will reach the detector. Third, one needs to ensure that all the microscope hardware (PMT, cables, power supply, pre-amp) is functioning properly. This can be done by exchanging components from the unaffected working fluorescence channel. This should first be done all at once, and if the channel becomes functional, individual components can be swapped back and forth to identify the problem component.

3) Potential Problem: I cannot see either fluorescence channel.

Potential Solution: First, the computer hardware should be tested. Use the scope control software (i.e. ScanImage) and/or the NI Max software panel to test that your NI boards, cables and breakout boxes can receive signals. A common AA battery and two wires can be used to test if ScanImage or the NI Max software can detect a 1.5V voltage signal. Second, the builder needs to ensure that all the fluorescence optics are in the right location and proper orientation. For example, if the emission filters are in the wrong place (e.g. red is located where the green should be, and the green is where the red should be), very little fluorescence will be detected on either channel. Third, all the microscope hardware (PMT, cables, power supply, pre-amp) needs to be checked. An oscilloscope can be used to check that the PMTs are sensitive to light. One can hook up the PMT signal cable coming from the pre-amp into the oscilloscope and carefully expose the PMT to small amounts of light. Additionally, a voltmeter can be used to ensure that all the cables are conducting current and are not shorted.

4) Potential Problem: One of my fluorescence channels is bright on one side and dark on the other, yet the other channel looks evenly illuminated.

Potential Solution: The most likely cause for this is either the secondary dichroic is not at 45 degrees, or the aspheric PMT lens is crooked on the affected channel (i.e. not sitting flush against the emission filter).

5) Potential Problem: My scanning mirrors are not scanning at all.

Potential Solution: Use an oscilloscope to test if the control software (i.e. ScanImage) can output appropriate voltage signals to control the mirror. If present, one can examine the amplitude as well as the frequency of the voltage signals for accuracy. One should also test to see if one can detect the difference between saw-tooth and bi-directional scanning. If everything looks good, the problem may be with the scanning mirrors themselves. If scanning voltage command signals are not present, the NI Max software panel can be used to test if the boards can output different types of signals (steps or sine waves) to be detected by an oscilloscope. If the board checks out then the problem is likely the control software. However, if problems are detected in the NI hardware, another board, cable and breakout box can be tested if available, and if possible another computer should be tested. These steps will hopefully identify whether the problem lies in the scanning mirrors themselves, the software control or the hardware control of the scanning mirrors. Also see the related problem/solution below concerning scanning problems.

6) Potential Problem: My scanning mirrors are scanning intermittently or erratically.

Potential Solution: In wiring up TIMAHC one has to make many cables. The hardest to make is the cable that sends the voltage signals from the breakout box to the scanning mirror servo driver board. Here, the bare ends of four wires (a signal and a ground from each BNC output for the x and y mirror command) need to be soldered onto small metal female pins that insert into a six slot plastic connector (two middle slots are not used). This connector connects the wires/pins to the servo board. The metal female pins do not insert easily (deep enough) into the plastic connector and, if done poorly, a bad connection could result causing intermittent scanning behaviour. Take a fine Allen key and a small hammer, place the fine tip of the Allen key on the top of the female pin and gently hammer the female pin (that is soldered to the wire) deep into the plastic connector until the pin and plastic are flush with each other at the end that connects to the servo board.

7) Potential Problem: After imaging for several minutes, the image suddenly becomes wavy and distorted.

Potential Solution: First, one needs to make sure that you are not scanning too fast for your zoom factor (refer to the Considerations and Limitations section of the main article). If scanning speed is not the issue, check to see if the scanning mirror servo board is over heating. Proper heat sinking and subsequent cooling of the servo board is critical to its performance. One needs to confirm that a very thin layer of thermal paste (scraped on) was applied between the surfaces of the heat sink and the servo driver. Too much paste can act like an insulator, causing heat to be retained on the board. Confirm that the screws are finger tight which fasten the heat sink to the servo board. A firm connection between the surfaces is important. Finally, ensure that the computer fan mounted above the sink is still working.

8) Potential Problem: The core of my point-spread function is large and not nearly diffraction limited.

Potential Solution: One needs to ensure that the beam is collimated entering the back aperture of the objective lens. If necessary (!!safety goggles necessary!!) one can redirect the beam after it passes through the tube lens and project it away from the scope to a safe location (!!caution!!). This increased distance will allow greater accuracy in assessing collimation. Additionally, ensure the diameter of the beam slightly overfills the diameter of the back aperture of the objective lens. If the beam diameter is too small (i.e. under filled back aperture), this will result in a numerical aperture drop and larger resolution limit. If so, different optical table achromatic lenses will be needed with a greater expansion ratio (greater ratio between the focal lengths of the lens). In the TIMAHC configuration presented, the max beam diameter is ~18.5mm due to the size of the scanning mirrors (5mm) and the expansion ratio given by the scan lens and tube lens (3.7x).

9) Potential Problem: My point-spread function is not circular but oblong.

Potential Solution: First ensure the beam is traveling along the table and up through the scope at right angles and is traveling down the optical axis well (see problem/solution 1 for optimizing the beam path and optical axis). If after optimizing the beam path the problem still persists, one needs to check whether the detector sub-assembly is level and centered properly. Mounting and aligning the detector sub-assembly well is the most difficult assembly task when building TIMAHC. If the detector assembly is even slightly askew, the point-spread function will likely suffer.

10) Potential Problem: I see an artifact/aberration in my image timed with the rate of scanning yet the scanning mirrors are working well.

Potential Solution: Ensure that the height or position of the objective lens along the optical axis lies on a conjugate plane to the scanning mirrors when imaging. To do this, first move the objective lens to the height it would be for imaging the sample. Next, start scanning and use the anti-stokes beam illumination card to see how the height of the back aperture of the objective lens corresponds to where the scanning-induced movement in the beam is minimally observed. Where there is the least movement in the beam is the optimal position for the back aperture of the objective lens. At a height above or below this optimal position, the scanning-induced motion in the beam should be easily detected. If an offset is detected (i.e. the back aperture of the objective lens and the conjugate image are not at the same position in the optical axis), the height of the stage and consequently the condenser assembly will need to be adjusted. If the discrepancy is so large that a stage height adjustment cannot solve the problem due to limits imposed by the 1 inch travel range on the z slider, then the height of the entire detector sub-assembly can be adjusted. However, if the detector sub-assembly is moved up or down the optical rail, subsequent adjustments to the stage height and condenser sub-assembly must follow.

11) Potential Problem: I cannot get my condenser to center into my field of view. Or, when I try to setup Koehler Illumination, I cannot center a mostly closed field iris in my image.

Potential Solution: The most likely source of this problem is an optical axis mismatch between the condenser sub-assembly and the detector sub-assembly. TIMAHC is design so that the centers of all the optical elements on the front of the main optical rail align in the z direction (i.e. the optical axis). Two sources of optical axis mismatch are: 1) the detector sub-assembly was not mounted accurately (see tips for building and mounting the Detector sub-assembly); 2) the optical axis of the condenser sub-assembly is not in the correct location (refer to model for the correct distance).

12) Potential Problem: When I try to setup Koehler Illumination, I cannot get my field iris in focus.

Potential Solution: An important variable for bringing the field iris into focus (conjugate to the image plane) is the height of the condenser lens relative to the tissue bath. In TIMAHC it may be difficult to get the condenser lens high enough, rather than low enough. This can be adjusted coarsely by sliding the entire condenser sub-assembly up the main optical rail, or very finely using the z translator that is attached to the condenser lens. However, if the condenser lens is as high and as close as it can be without touching the glass of the tissue bath with the field iris is out of focus, one can try making small adjustments to the height of the field iris by sliding it up and down the cage rods. Adjusting the height of the field lens can also be tried but there is less range in sliding this part up and down the cage system.

13) Potential Problem: When I look at my transmitted image on the IR-1000 camera, the image is a small bright dot OR the image is smaller than my monitor screen with warping (vignetting) around the edges, OR the magnification of the image is too high (cell too large) and is grainy looking.

Potential Solution: This problem occurs when the lens in between the sliding cube and the camera (in the scanning sub-assembly) is either not positioned properly or is the wrong focal length. If the lens is too far from the camera or of too short a focal length, the image will be a small bright dot or appear smaller than the monitor screen with warping around the edges. If the lens is too close to the camera or of too long a focal length, the magnification of the image will appear too large and may be grainy looking. Either changing the position of the lens or changing to a different focal length lens can help to solve this problem.

14) Potential Problem: When I look at my transmitted image on the IR-1000 camera, the image is clearly not centered because one edge is black OR is not centered because it does not match the fluorescence image I get generated by my PMTs.

Potential Solution: This problem can occur when the mirror in the sliding cube (in the scanning sub-assembly) is not mounted at 45 degrees. One can rotate this optic while looking at your transmitted image to see if it can be optimized. Also, the appearance of a black edge could be a partly closed field iris in the condenser sub-assembly that is not centered properly.

15) Potential Problem: I cannot see my under-stage transmitted PMT image.

Potential Solution: Check the orientation of the beam splitter cube at the bottom of the condenser sub-assembly. If incorrectly orientated, the transmitted Ti:Sapp light will not reach the under-stage PMT.

If this is not the problem, one needs to check the PMT hardware. A voltmeter can be used to check whether your cabling is free of shorts. An oscilloscope can be used to ensure the PMT is sensitive to light. Next, the imaging software (i.e. ScanImage) and/or the NI max software can be used to ensure the computer/software can receive signals. As above, an AA battery with two wires can be used to generate a 1.5V signal for testing.

16) Potential Problem: I cannot see my transmitted image generated by 940 nm LED at all.

Potential Solution: Check the orientation of the beam splitter cube at the bottom of the condenser sub-assembly. If incorrectly orientated, the LED light will not be reflected up towards the sample. Also check that the LED is providing an output. The eye cannot see 940 nm light. The NIR illumination card can be used to see if you LED is working. Finally one needs to be certain that the IR-1000 camera is sensing light by waving a flashlight near it and confirming light detection on the monitor.

Citations

1. Cole RW, Jinadasa T, Brown CM (2011) Measuring and interpreting point spread functions to determine confocal microscope resolution and ensure quality control. Nat Protoc 6: 1929–1941. doi:10.1038/nprot.2011.407.

Supplementary Figure 1: **Detector sub-assembly mounting alignment aid**. Close up of the detector sub-assembly (PMTs and objective lens removed) and the bottom aspect of the scanning sub-assembly. Parts coloured in blue (CP02, ER6 x 2, LCP02) are the mounting alignment aid. To add the alignment aid the tube lens should be removed from the scanning sub-assembly. The bottom of the CP02 plate should touch the top of the primary dichroic cube such that the back aperture of the objective lens will be approximately 200 mm from the middle of the tube lens. Importantly, this position is when the z slider has been descended almost to its full extent (the imaging position, not when the objective lens has ascended for tissue loading or pipette approach). The tool is necessary to mount the detector sub-assembly level on the optical axis and at a desired angle to create space for micromanipulator access to the tissue bath.
